# In situ potassium and hydrogen ion exchange into a cubic zirconium silicate microporous material

**DOI:** 10.1371/journal.pone.0298661

**Published:** 2024-03-21

**Authors:** Jason Lively, Aaron J. Celestian

**Affiliations:** 1 On Market Technical, Pharmaceutical Technology & Development, Operations, AstraZeneca, Coppell, TX, United States of America; 2 Department of Mineral Sciences, Natural History Museum of Los Angeles County, Los Angeles, CA, United States of America; Saveetha Institute of Medical and Technical Sciences: Saveetha University, INDIA

## Abstract

The selective separation of ions from aqueous systems, and even in the human body, is a crucial to overall environmental management and health. Nanoporous materials are widely known for their selective removal of cations from aqueous media, and therefore have been targeted for use as a pharmaceutical to treat hyperkalemia. This study investigated the detailed crystallographic molecular mechanisms that control the potassium ion selectivity in the nanoporous cubic zirconium silicate (CZS) related materials. Using time-resolved *in situ* Raman spectroscopy and time-resolved *in situ* X-ray diffraction, the selectivity mechanisms were determined to involve a synchronous cation-cation repulsion process that serves to open a favorable coordination bonding environment for potassium, not unlike the ion selectivity filter process found in potassium ion channels in proteins. Enhancement of ion exchange was observed when the CZS material was in a partial protonated state (≈3:1 Na:H), causing an expansion of the unit-cell volume, enlargement of the 7 member-ring window, and distortion of framework polyhedra, which allowed increased accessibility to the cage structures and resulted in rapid irreversible potassium ion exchange.

## 1. Introduction

The cubic zirconium silicate (CZS) materials used in this study can be considered a part of the porous heterosilicate family, a group that resembles natural microporous minerals that have zeolite-like properties. Ion exchange in zeolites and related microporous materials are important for industrial and environmental applications and have broad-ranging applications, including water purification [1], gas separation [2], catalysis [3], and heavy cation sequestration [4] from aqueous solutions. Even more recently, there has been a new drive to utilize these materials in medicine.

The partially protonated cubic zirconium silicate material, CZS-(Na,H), has been studied in human clinical trials in hyperkalemic (*>*5.1 mEq/L of K in blood) patients [5–9]. The clinical trials had shown to bring K^+^ in the blood back to normal levels within only a few hours. However, the crystallographic properties, and the ion exchange mechanisms, of the CZS materials have not been previously determined.

This study focused on determining the structural changes during the *in vitro* ion exchange process *in operando* on one of the first inorganic crystalline materials to be approved by regulatory agencies. The materials investigated were the as-synthesized sodium form of the cubic zirconium silicate (CZS-Na), also known as ZS9 or ZS-9, and the partially H^+^ exchanged form (CZS-Na,H), marketed as Lokelma®. Time-resolved X-ray diffraction and time-resolved Raman spectroscopy (both performed *in situ*) were used to collect data during the K^+^, H^+^, and Na^+^ exchange process in the CZS family of materials. By using identical experimental conditions between X-Ray Diffraction (XRD) and Raman, experimental molecular trajectory models from over 2,000 crystal structure refinements (from XRD, long-range order) coupled with molecular changes obtained from time-resolved Raman spectroscopy (short-range order) were correlated to help determine the ion selectivity mechanisms. Supporting data were also collected to confirm H_2_O content in the structure using simultaneous thermogravimetric and differential scanning calorimetric analyses.

### 1.1. Crystal Structure of CZS

The CZS-Na material (Na_2_ZrSi_3_O_9_ ·2.5(H_2_O)) is a cubic cyclosilicate (Pa¯3, a = 12.7416(1) Å) with zirconium octahedra bonding to the silicate 6MR (member rings) to form a 3D heterosilicate framework and was provided by the pharmaceutical company AstraZeneca [10].

Crystallographic information files are provided in the supplementary information for the suite of CZS crystal structures [11]. All CZS materials have the same Pa¯3 space group, but have different extra-framework cations and cation ratios within the cages and channels. The compound labeling scheme suffix has the starting cation listed first, followed by the first exchange cation, and then the third exchanged cation (when applicable). End-member compositions are listed with only one cation in the suffix, and all materials were ion exchanged from the as-synthesized CZS-Na (Table 1).

**Table 1:** Description of materials in this study. Formula determined by Rietveld refinement. H by difference calculation; values have not been verified by chemical analysis.

Abbrev.	Description	Formula
CZS-Na*^,1^	As-synthesized CZS material	Na_2_ZrSi_3_O_9_ ·2.5(H_2_O)
CZS-K^2^	Maximal K exchange into CZS-Na	K_2_ZrSi_3_O_9_ ·H_2_O
CZS-H^3^	Maximal H exchange into CZS-Na	H1.3Na0.7ZrSi3O9 ·3H2O
CZS-(Na,H)*^,4^	Partial hydrogen exchange into CZS-Na	H≈0.5Na≈1.5ZrSi3O9 ·2.8H2O
CZS-(Na,K)^5^	Partial K exchange into CZS-Na	NaKZrSi_3_O_9_ ·1.5H_2_O
CZS-(Na,H,K)^6^	K exchange into CZS-(Na,H)	Na0.4K1.6ZrSi3O9 ·H2O

* Indicates commercial samples. Structure deposited in the Cambridge Structural Database under numbers: ^1^ 2301919, ^2^ 2301923, ^3^ 2301920, ^4^ 2301921, ^5^ 2301918, ^6^ 2301922

**Fig 1:**
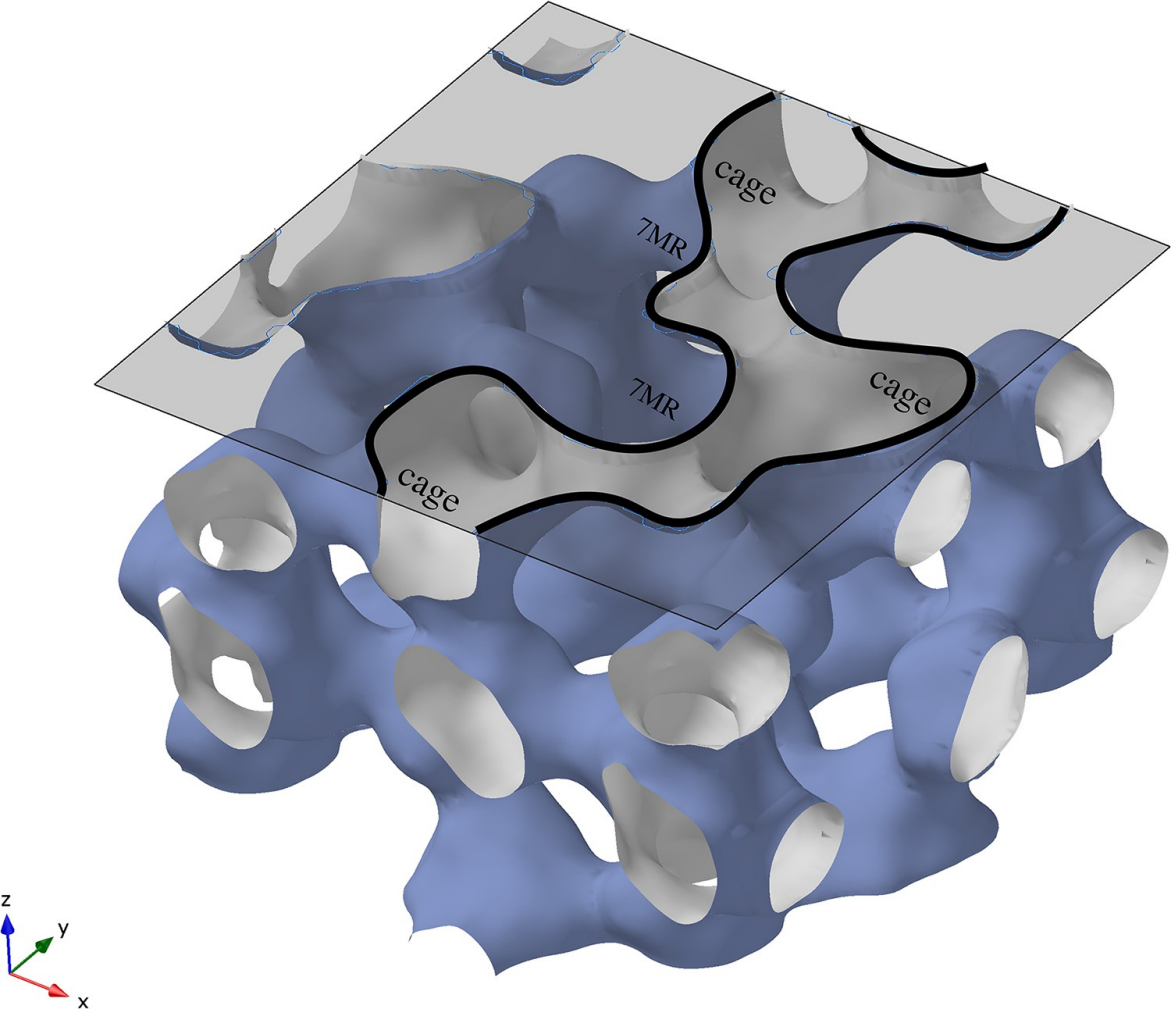
Solvent accessible map of the CZS material. White area shows internal void volume with cages and 7MR windows labeled. Cross-section of a channel is outlined in black. All atoms removed for clarity.

**Fig 2:**
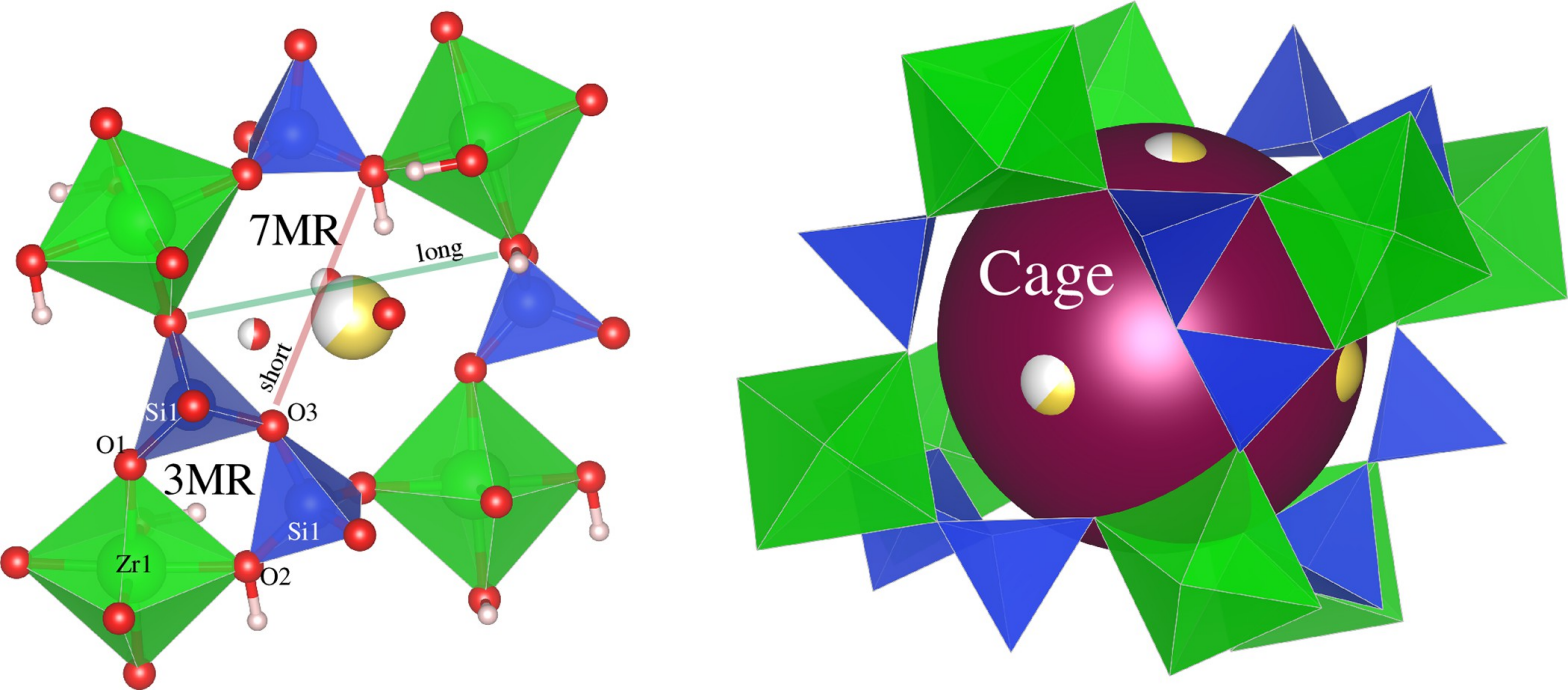
Illustrations of the 7MR and 3MR with calculated H^+^ positions based on bond valence calculations (left), and the large cage that is created at the intersection of the 7MR channels (shown as purple sphere). Na^+^ positions (yellow) are found at the windows of the changes, which are the centers of the 7MR. Calculated OH^−^positions point toward the center of the 7MR. Longest and shortest 7MR dimensions indicated by solid lines. Crystal structures drawn using VESTA [13].

All the CZS materials can be described as being built of hexagonally close-packed cages of SiO_4_ and ZrO_6_ groups (Figs 1 and 2). These cages are formed at the intersection of the three 7MR channels, where the three channels are aligned parallel to the a-, b-, and c-axis directions, and contain all extra-framework cations and H_2_O. The cage is built from both 3MR and 7MR secondary building units whereby atomic access to the CZS cage (volume of 207 Å^3^) should be limited by the diameter of the 7MR (see Fig 1).

For comparison, the mineral sodalite is a porous aluminosilicate zeolite framework, having a crystal structure built on a series of hexagonally close-packed *β*-cages [12]. Sodalite *β*-cages have an approximate volume of 270 Å^3^ with 6MR windows that access the cage. The ionic diameter of the sodalite 6MR window is approximately 5.1 Å. Despite CZS-Na having smaller cage volume than sodalite, the CZS-Na material is likely to be more permeable for cation exchange as the 7MR cage access windows are larger (up to 6.5 Å, as measured from sites O2 to O2).

The 3MR in the CZS materials is shown in Fig 2 and is formed from Si1-O1-Zr1-O2-Si1-O3 connectivity (refer to crystallographic information file for site labeling). In the CZS-(Na,H) and CZS-H (Fig 2) form, this 3MR contains OH groups that are likely located near the O1 site (based on bond valence sum analysis, see supplementary information). Neutron diffraction data would be needed for H/D site confirmation, and these experiments are being planned for the future. The Si-O-Zr bridging O is also the site for Na^+^ bonding and K^+^ bonding to the framework.

The 6MR in CZS is solely composed of SiO_4_ forming rings of composition Si_6_O_18_, and is formed by the join of three 3MR. Although the 6MR does not host any of the charge balancing cations, the geometry of the 6MR was significantly affected by the host-guest cation type and position in the 7MR and cages.

The 7MR is the largest confining ring-opening that connects the hexagonally closest packed array of CZS-cages. The 7MR (Fig 2) is composed of four SiO_4_ and three ZrO_6_ groups with an average long-axis and short-axis of approximately 6.5 Å (between O2 and O2) and 5 Å (between O3 and O1), respectively. This O3···O1 distance is further reduced in the CZS-(Na,H) structure as the H sites forms hydroxyl groups on O1 resulting in an O3···H distance of 4.2 Å. The H is located in the window of the 7MR on the O1 site (Fig 2).

Bond valence sum (BVS) calculations [14–16] were used to predict the H coordination to the ZrO_6_ groups in CZS-(Na,H) (see supplementary information). The Zr-O1 bonds have lower BVS as compared to the Zr-O2 bonds. The lower BVS for the Zr-O1 is an indicator that the H sites are bound to the O1 in the structure. These calculations were also used to determine the BVS for Na in the CZS-Na structure (BVS_Na_ = 0.70), which is 30% under-bonded (a BVS difference of ± 15% is generally acceptable). If K^+^ were to directly replace Na^+^ in the same crystallographic site, without distortion of the framework, then BVS_K_ = 1.7, which is 70% over-bonded. This BVS calculation may indicate that the 7MR needs to distort to accommodate the in-going K^+^.

## 2. Methods

### 2.1. Ion exchange

The experimental setup of this study is similar to previous research performed for *in situ* ion exchange studies [17, 18] and is briefly described here. The commercial CZS-(Na,H) and as synthesized CZS-Na material were separately loaded into their own custom corundum tubes from Saint-Gobain (inner and outer diameters are 1.0 mm and 1.6 mm, respectively), and held in place in the center of the tube by glass wool filters (see supplementary information for details). The crystalline powder is centered just below a lens in the corundum tubing.

This lens was made using a 13 mm diameter polishing tool to generate a concave depression. This lens reduces the thickness of the corundum tube (reducing light absorption), and also served to refocus some of the scattered Raman light back into the microscope objective to obtain better signal:noise ratios. Raman scattering from the corundum served as an internal standard line-reference to calibrate for any spectral drift. The corundum tube is then positioned on the Raman microscope stage, and then connected to plastic tubing that is attached to a programmable peristaltic pump. Data is first collected on a dry sample, then data is collected on a wetted (using deionized H_2_O) sample. After a Raman spectrum is collected on the wetted sample, the H_2_O bottle is replaced with a bottle containing the electrolyte solution, and the data collection software is programmed to run autonomously. There is a natural concentration gradient developed at the water-electrolyte interface that is present in the plumbing. As a consequence of this concentration gradient, transitions in the peak evolution profile caused by ion exchange, for both the Raman and XRD data, are smoothed and not abrupt. This results in slight model miss-fitting of exchange curves for kinetics analyses. This study does provide kinetic rates of ion exchange, but the major focus of this work is the molecular controls of cation exchange selectivity, and these molecular motions have been shown to be independent on the concentration gradient within the tubing [19].

The choice of electrolyte concentration was based on several criteria. A 0.01 M solution of NaCl or KCl was used as the electrolyte, which is a concentration approximately 100x higher than in normal blood. Typical blood will have a Na concentration of ≈ 3.10 mg/L (of NaCl) or ≈ 0.00005 M. Based on previous work [17, 19, 20], such low concentration solutions would be difficult to prepare with precision, and also these very low molarity solutions would potentially slow the ion exchange processes beyond allocated experiment beamtime at the synchrotron.

**Fig 3:**
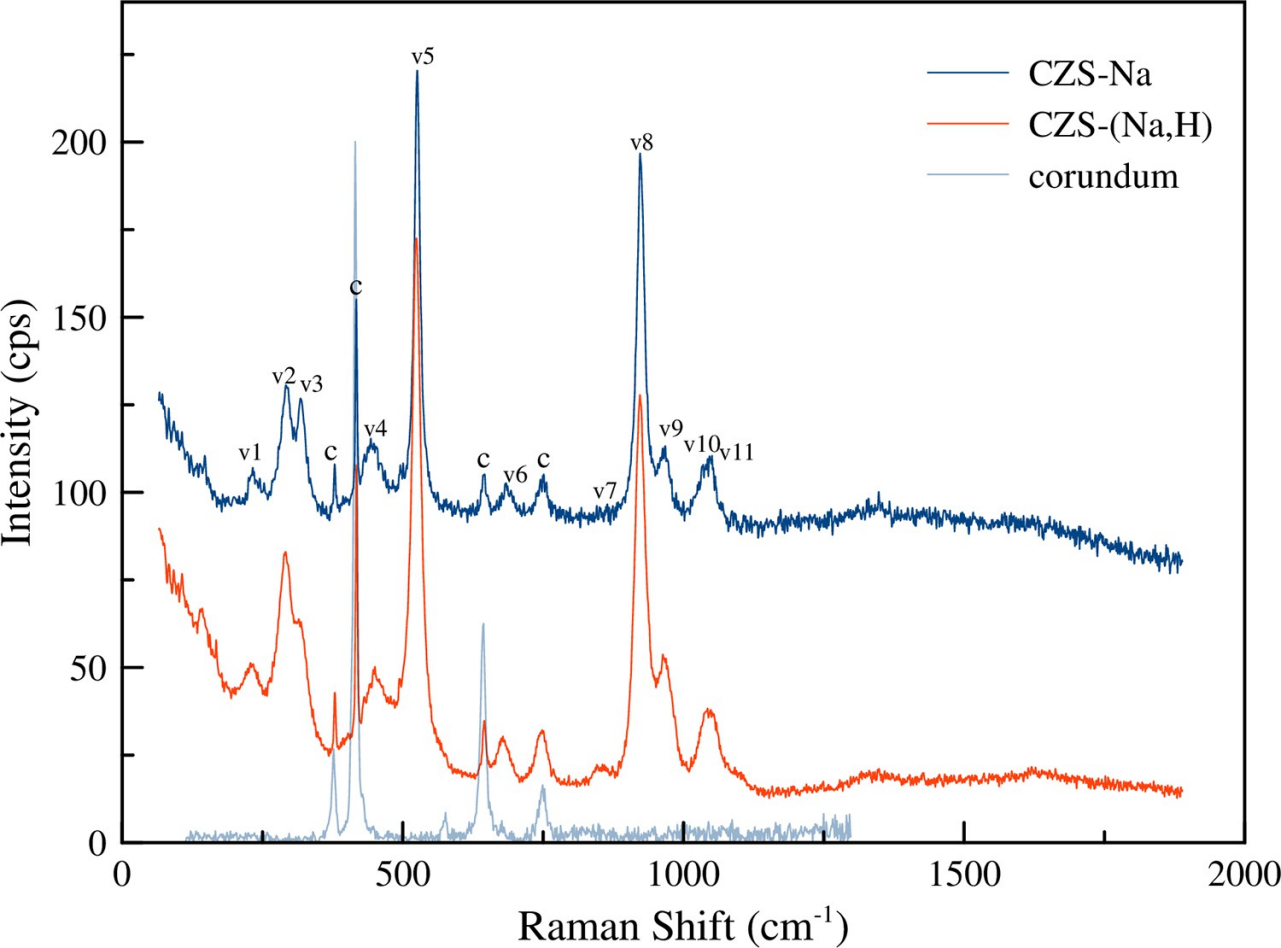
Raman spectra for CZS-Na (blue), CZS-(Na,H) (orange), and corundum (light blue). Peaks labeled ‘c’ are from the optically transparent corundum tubing. The corundum peaks are used to correct for spectral drifts during time-resolved experiments.

Ion exchanged solutions of NaCl (0.01 M), KCl (0.01 M), and HCl (0.01 M) in deionized water were used. These higher concentrations will ensure *in situ* ion exchange studies will show a complete exchange process in a reasonable experimental timeframe (≈4 hrs). Raman spectra for CZS-Na, CZS-(Na,H), and corundum are shown in Fig 3. The corundum Raman peaks were used as an internal standard to correct for any spectral drift during the experiment. Having an internal standard allowed for greater confidence in small peak shifts over the course of the experiment.

### 2.2. Raman microscopy

The time-resolved Raman microscopy data were collected with a Thermo-DXR Raman microscope with a 780 nm near-infrared laser set to a power of 14 mW at the sample. The exposures for all experiments in this study were 10 sec. 3x using an 1800 gr/mm diffraction grating. For all Raman experiments, a total of 400 spectra were collected. Peak and background fitting were performed in the software package MagicPlotPro (fitting procedures are described in the supplementary information).

#### 2.2.1. Raman mode analysis

**Fig 4:**
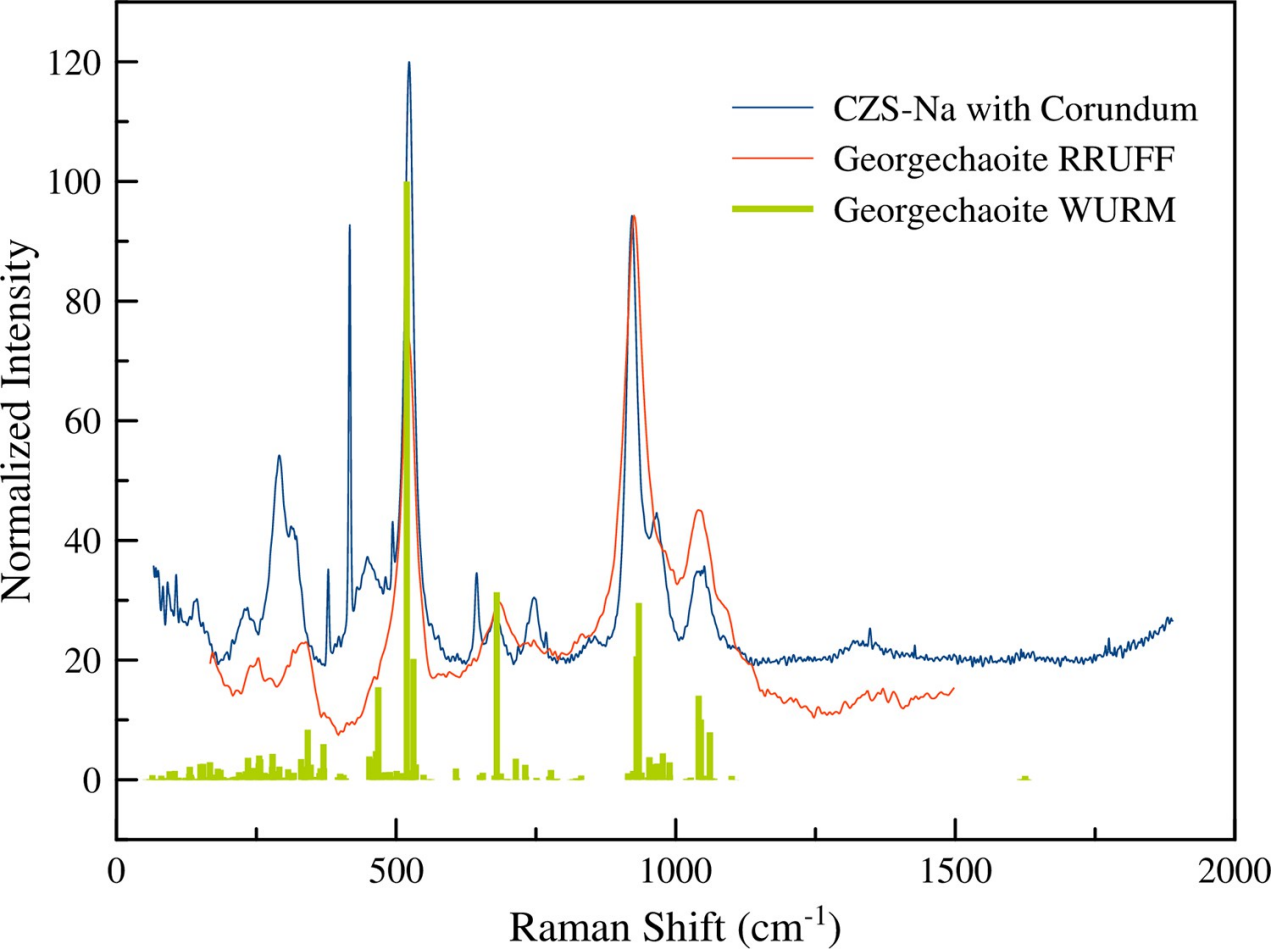
Comparison of Raman spectra for the CZS-Na material (this study), georgechaoite data from RRUFF.info, and calculated georgechaoite from WURM.info.

Raman modes were determined by comparing the DFT (density functional theory) calculations of the georgechaoite (a zirconium silicate mineral with 3MRs) NaKZrSi_3_O_9_ ·2H_2_O (retrieved from RRUFF [21] and WURM [22] databases, ID’s R100082 and W000239, respectively) (Fig 4) to CZS-Na. The Raman spectra for zirconium silicates with 3MR CZS-Na, georgechaoite, hilairite, and gaidonnayite all have strong similarities. Of these minerals, there exist small shifts of the 520 cm^-1^ and 920 cm^-1^ peaks to lower wavenumbers for georgechaoite, which was likely due to the longer Zr-O bonds, distortion of the 3MR, and larger unit-cell for georgechaoite. The DFT calculations show that the most intense peaks for georgechaoite (and therefore CZS-Na) indicate that the 920 cm^-1^ peak (and peaks at higher wavenumbers) is a result of stretching along the Si-O-Si-O 6MR ring, known for Si-O asymmetric stretches [22] (e.g., beryl, benitoite, and others), and the 520 cm^-1^ peak is the symmetric stretch mode of the 3MR. The 686 cm^-1^ peak is the Si-O-Si bend. In addition, comparative analyses were made using Raman spectra for gaidonnayite [23] and 3MR’s from the mineral zorite [24], both indicated that the 520 cm^-1^ peak is from 3MR stretching. Other peaks in the Raman spectrum were not well resolved and unreliable to model without DFT calculations specific to the CZS material, and therefore not included in the analysis of this study, the remaining areas in the Raman spectrum can be explored on the WURM.info database. The complimentary Raman-XRD analyses in this study indicated that changes in the 520 cm^-1^ Raman peak is a useful indicator to understanding ion exchange processes in CZS.

### 2.3. X-ray diffraction

**Fig 5:**
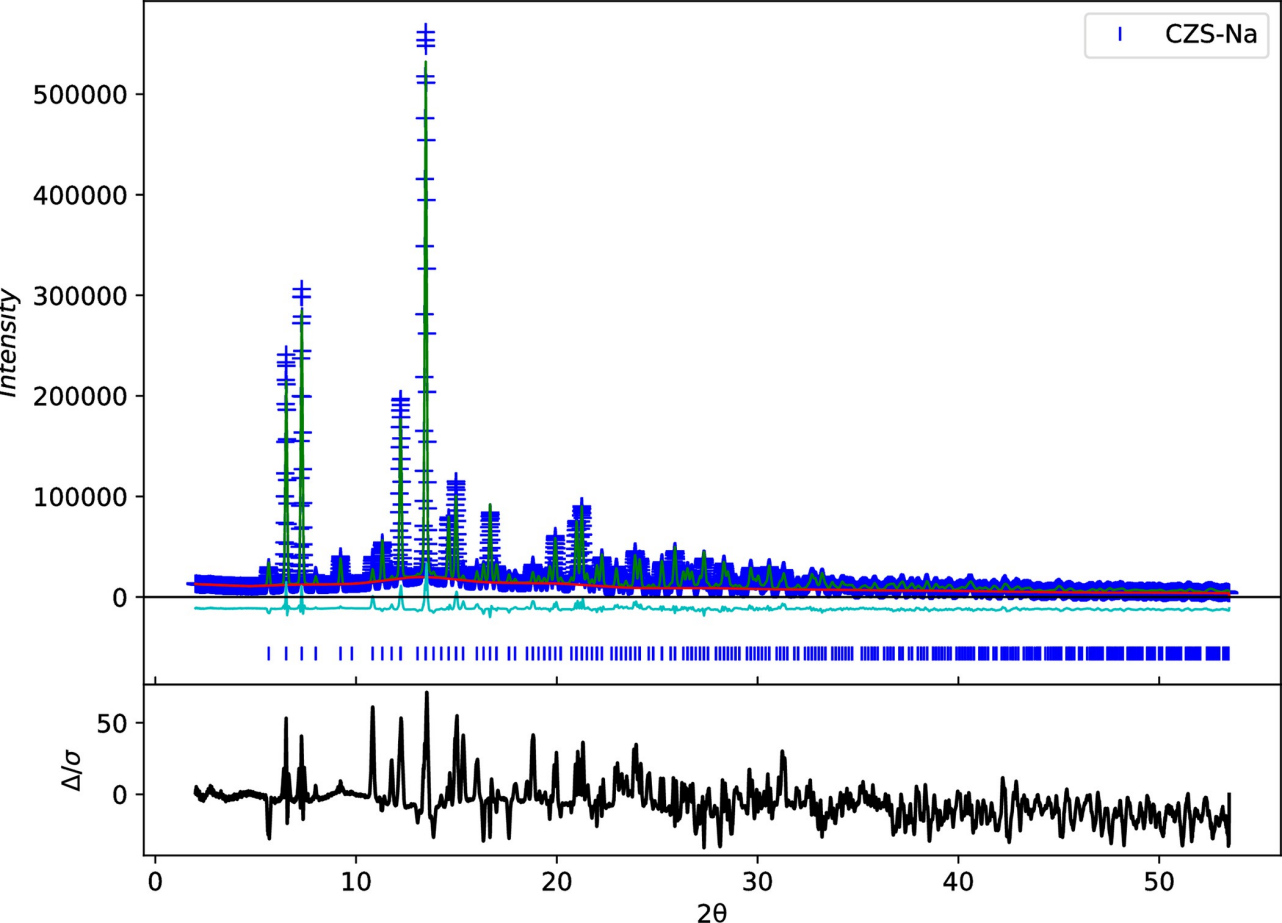
Example Rietveld refinement plot for CZS-Na from synchrotron data. Data point are blue ‘+’, calculated diffraction pattern is green solid line, background curve is red line, difference curve is cyan line, peak positions are shown as blue ticks, and Δ*/σ* curve is shown in black.

Both sealed-tube and synchrotron X-ray diffraction data were collected. *In situ* synchrotron XRD experiments in transmission-geometry were performed at beamline 17-BM of Advanced Photon Source, at the Argonne National Laboratory in order to monitor changes in the unit-cell parameters as the ion-exchange process occurred. The *in situ* cell was adopted from the Raman flow through cell design, but used a polyimide tube instead because of its X-ray transparency properties. The flow was controlled by a syringe pump. The wavelength used was 0.72768 Å, and data were collected to a minimum d-spacing of 1.244 Å with the sample detector distance set to its maximum (1300 mm). GSAS-II [25] was used for all data treatments including image integration, unit-cell refinements, and atomic structure refinements (See example refinement in Fig 5). Sealed-tube XRD *ex situ* data were also collected on a theta-theta geometry Proto AXRD and Rigaku Miniflex-II diffractometer (Cu k*α*) after the thermal analysis experiments. The PXRD scan parameters were set to scan from 5° to 55° 2*θ* at 0.5°/min, which was sufficient for unit-cell refinements.

## 3. Results

### 3.1. K exchange into CZS-Na

**Fig 6:**
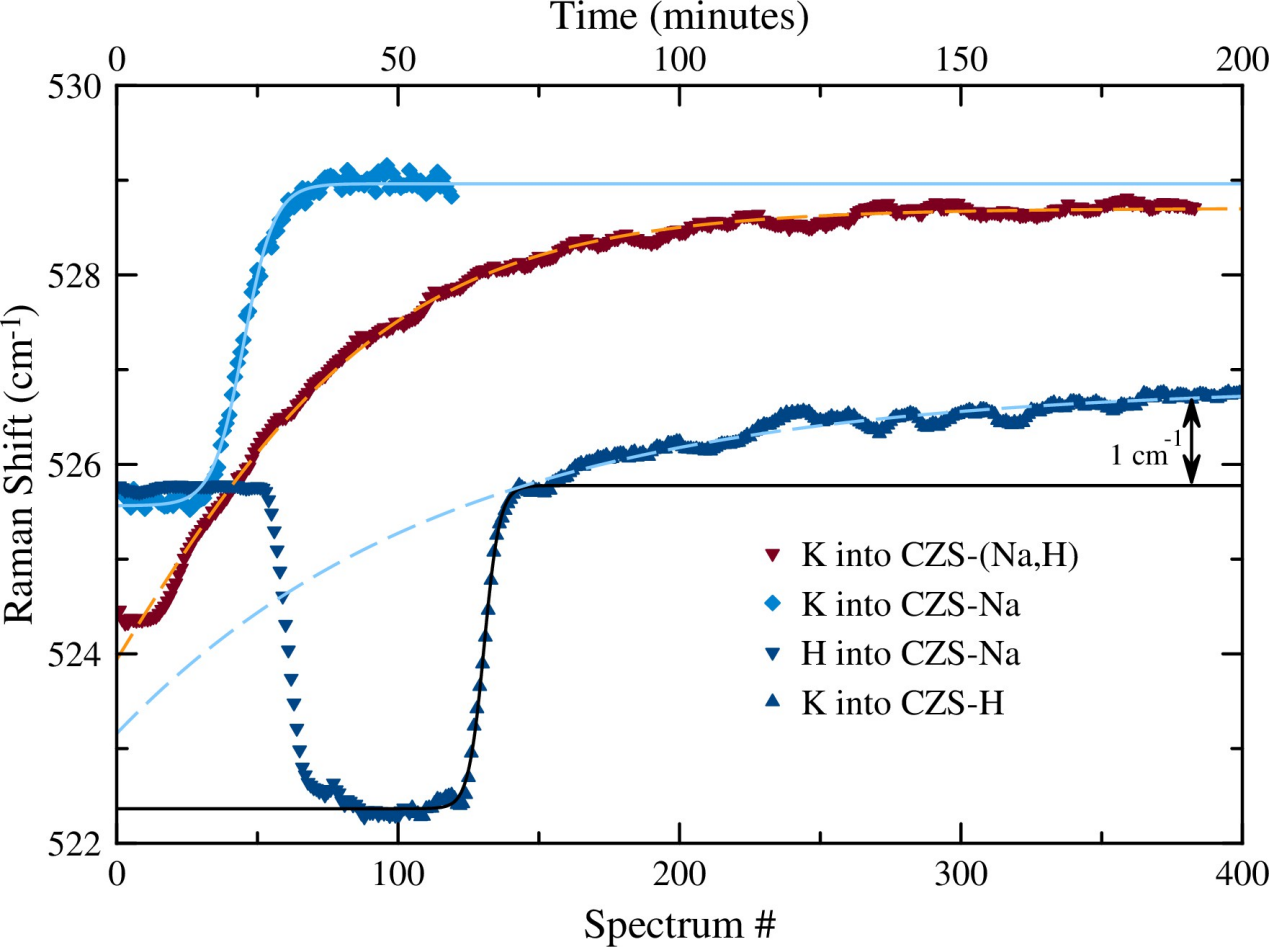
Peak evolution for the v5 peak (Si-O-Si bend in the 3MR) as observed from time-resolved Raman spectroscopy for the K into CZS-(Na,H) (red triangle down), K into CZS-Na (light blue diamond), H into CZS-Na (dark blue triangle up), and K into maximal H exchanged (dark blue triangle down). Peak evolution data for the K into CZS-Na curve is shown incomplete for clarity. Dose-response models are shown as lines for each experiment (excluding H into CZS-Na for clarity). For the K into CZS-H, two models are shown: black for the first exchange step, and dashed light blue line for second exchange step. A single model dose-response model would not fit the entire K into full CZS-H exchange experiment.

**Fig 7:**
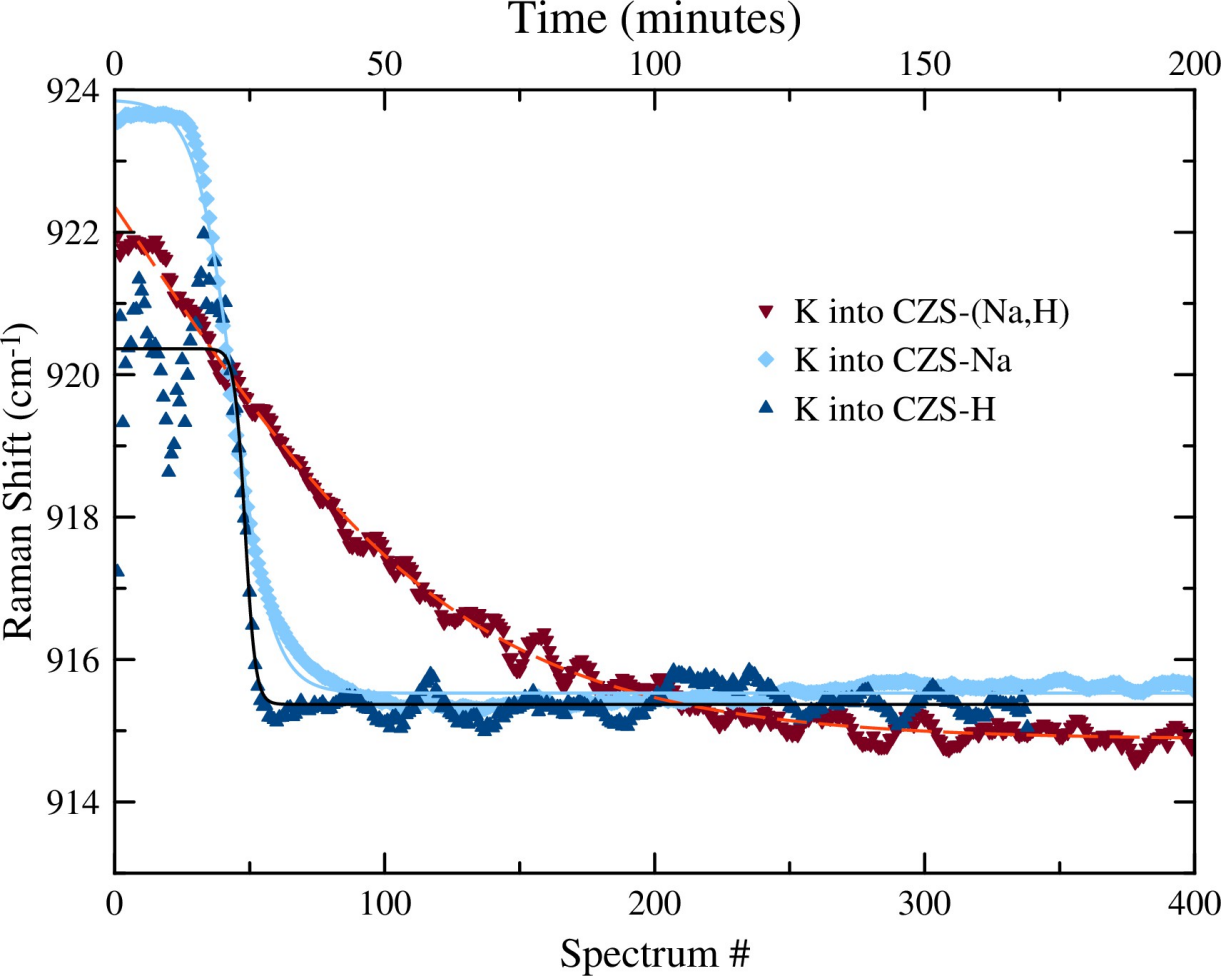
Plot of the v8 peak for K ion exchange into CZS-(Na,H), CZS-Na, and maximal H exchanged CZS-Na (CZS-H). For CZS-H, oscillations between time 0 and 25 minutes are within error of a peak centered at ≈ 920 cm^-1^ that is a result of poor peak fitting of the very low intensity v8 peak.

The time-resolved Raman spectroscopy data and results of the peak fitting for the v5 and v8 peaks are shown in Figs 6 and 7, respectively, and in Table 2. These data show a relatively rapid uptake of K^+^ starting around minute 20. The v5 peak shifts to higher wavenumber, while the v8 peak shifts to lower wavenumber (Table 2). The shift of the v5 peak may be explained by the high bond valence interaction between K^+^ and the O^2–^ on the 3MR sites, while the shift of the v8 peak indicates a lengthening of the Si-O bonds. The lengthening of the Si-O bonds indicates a greater electrostatic interaction between the framework O^2–^ and the new K^+^ inter-channel cation species. Lengthening of the Si-O bond was also observed in the XRD data. Raman spectroscopy alone cannot determine the location of the K sites, but based on previous discussion above, a probable location for the K^+^ is just outside of the 7MR window that would satisfy charge neutrality of the framework and bond valence of the K sites.

**Table 2:** Selected statistics for the v5 and v8 peak during ion exchange using dose-response model*. Unstable refinements and no peak information, are included (indicated by ‘-’).

	K → CZS-Na		H → CZS-Na		K → CZS-H		K → CZS-(Na,H)	
Parameter	v5	v8	v5	v8	v5	v8	v5	v8
Top (cm^-1^)	528.9	923.8	525.8	924.1	525.7	920.4	528.7	921.7
Bottom (cm^-1^)	525.5	915.5	522.4	921.5	522.3	915.3	524.6	914.9
Hillslope	0.18	-0.14	-0.36	-	0.36	-0.52	0.018	-0.016
EC50 (min)	22.6	22.01	30.4	-	65.25	-	≈12	≈12
Start (min)	12.5	9.8	25.2	-	59.8	-	≈6	≈6
Stop (min)	31.5	32.3	35.5	-	70.4	-	-	-

* $y=bottom+\frac{\left(top-bottom\right)}{1+10^{Log(EC50-X)HillSlope}}$

**Fig 8:**
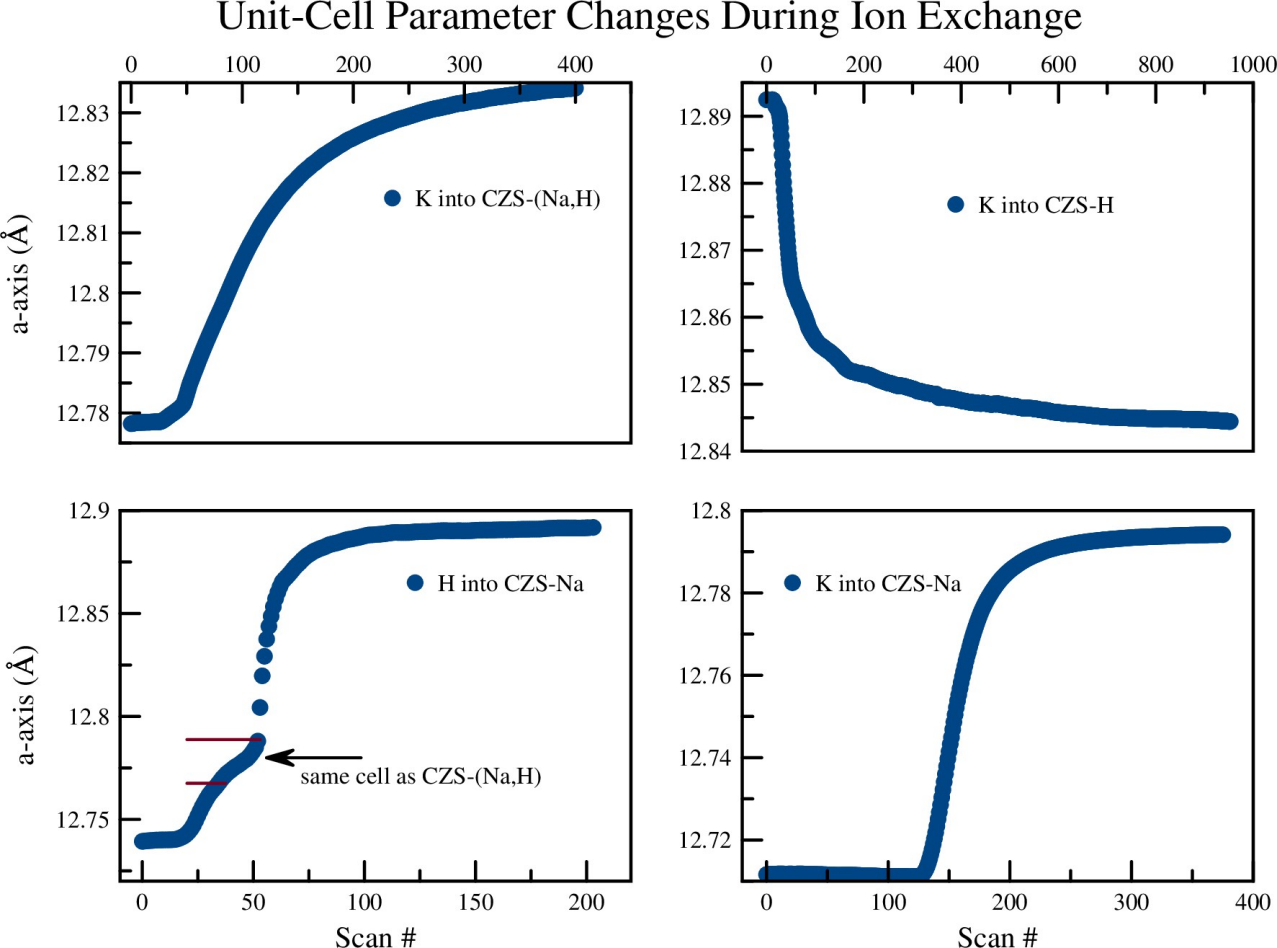
Changes in unit-cell parameters for the time-resolved *in situ* XRD synchrotron experiments. For the H into CZS-Na experiment, there was a change in the rate of unit-cell length increase as indicated by the boundary between the red horizontal lines. Near the top of this boundary, the unit-cell is the same as that for the commercial CZS-(Na,H) material (partial H^+^ exchanged CZS-Na). Data points are larger than calculated error values.

**Fig 9:**
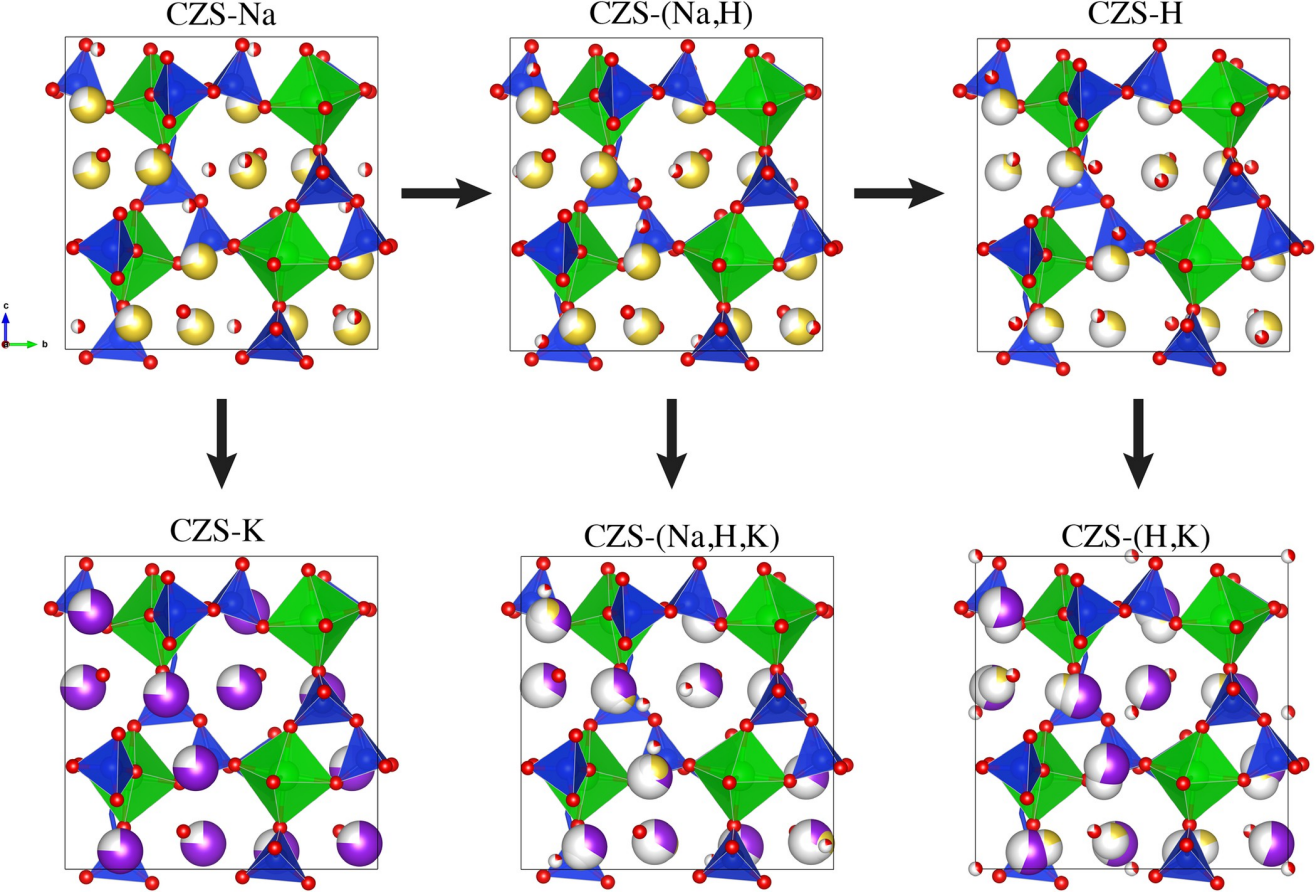
Crystal structures refined from XRD data, this study. Zr shown as green, Si shown as blue, O shown as red (H_2_O are shown as interstitial O), Na shown as yellow, and K shown as purple. Partially filled atomic sites reflect refined fractional occupancy values.

**Fig 10:**
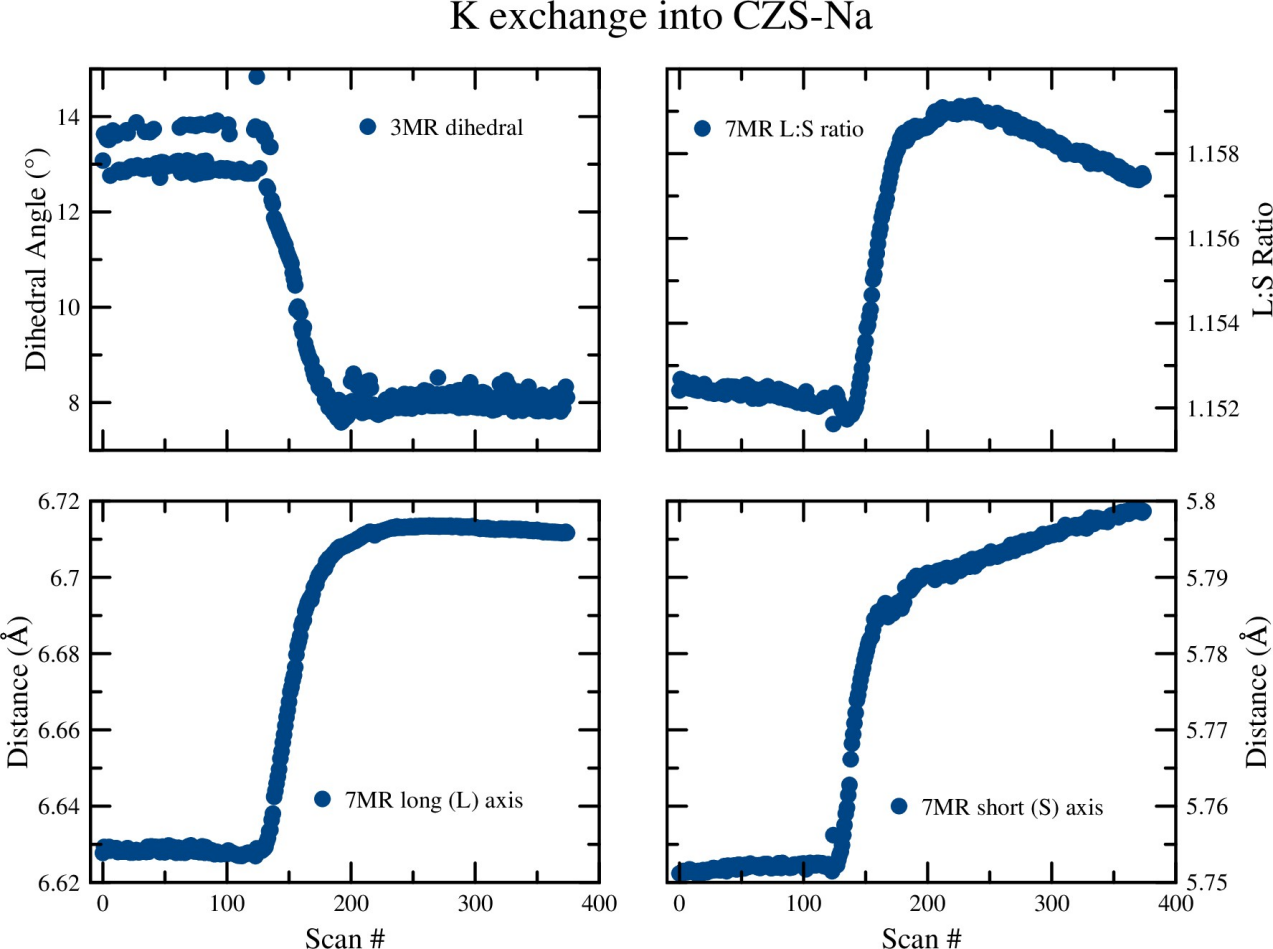
Analysis of crystallographic changes during K exchange into CZS-Na. Onset of the ion exchange process starts at approximately Scan# 130. Data points are larger than calculated error values.

From the synchrotron time-resolved diffraction data studies (summary in Table 3), unit cell parameters determined (Fig 8), crystal structures of all endmember and intermediate phases were refined (Fig 9), and the ring geometry (Fig 10) was tracked through time. During the K^+^ exchange into CZS-Na the disordered Na…Na distances (3.7Å and 4.5Å) and the disordered K…K distances (3.2Å and 4.0Å) are observed. These shifts are shown for the Na and K sites in Table 3. Select statistics for the v5 and v8 peak during ion exchange. Data for the v8 peak after H exchange were unstable and therefore not quantified (indicated by ‘-’) except for parameters ‘Top’ and ‘Bottom’. However, the average bond distance to framework O^2–^ increased from Na-O to K-O (2.59Å and 2.73Å, respectively). In addition, the torsion angle of the 3MR (Zr-O2-Si-O3) (Fig 10 decreased from structures CZS-Na (5.4°) to CZS-(Na,K) (4.6°). From Rietveld refinements, no excess unaccounted electron density was found in the center of the 7MR. Attempts were made to place K in the center of the 7MR, however, subsequent least squares refinements moved the K site 0.6Å out of the 7MR plane and into the cage.

**Table 3:** Final results from Rietveld crystal structure refinements. ^1^Distortion index (unitless); ^2^torsion angle; ^3^total bond valence sum (valence units).

	ZrO_6_ DI^1^	3MR (°)^2^	K BVS^3^	Vol. (Å^3^)	R, wR (%)
CZS-Na	0.0002	5.4	-	2068.57	6.98, 9.35
CZS-(Na,H)	0.0004	6.0	-	2074.23	4.83, 6.24
CZS-H	0.04	2.7	-	2142.52	7.31, 9.33
CZS-(Na,K)	0.02	4.6	0.80	2094.29	7.76, 10.48
CZS-(Na,H,K)	0.003	10.0	0.93	2119.10	5.56, 7.43
CZS-(H,K)	0.008	9.0	1.41	2114.00	7.76, 14.46

^1^Distortion index (unitless)

^2^torsion angle

^3^total bond valence sum (valence units).

### 3.2. H exchange into CZS-Na

The peak fitting results of the time-resolved Raman spectroscopy for the v5 and v8 peaks are shown in Fig 6 and Fig 7, respectively, and in Table 2. The H^+^ exchange into the CZS-Na material was rapid and significantly reduced the signal:noise ratio in the Raman spectrum, and resulted in poor peak fitting for all but the most intense v5 peak. Based on the v5 peak position and intensity, the exchange process took 10 min., compared to 19 min. for the K^+^ exchange into CZS-Na. The overall decrease in the intensity of the Raman signal may correlate to increased atomic positional disorder or increased strain on bonds. Loss of crystallinity was ruled out since the data from the XRD experiment showed no significant peak broadening or loss of overall intensity. The lattice vibrations at ≈ 300 cm^-1^ disappeared, the v5 and v8 peaks broadened. Raman scattering peaks for isolated or polymerized SiO_4_ or ZrO_6_ at 775 cm^-1^ and 200 cm^-1^ frequencies, respectively [26, 27] were not observed, that would indicate significant leaching of those molecular constitutes from the CZS-Na material. The widening of the full width half maximum (FWHM) for the CZS peaks in the Raman spectrum could arise from decreasing particle size, or increased local disorder, rather than dissolution of ZrO_6_ or SiO_4_ from the material [20, 27, 28].

**Fig 11:**
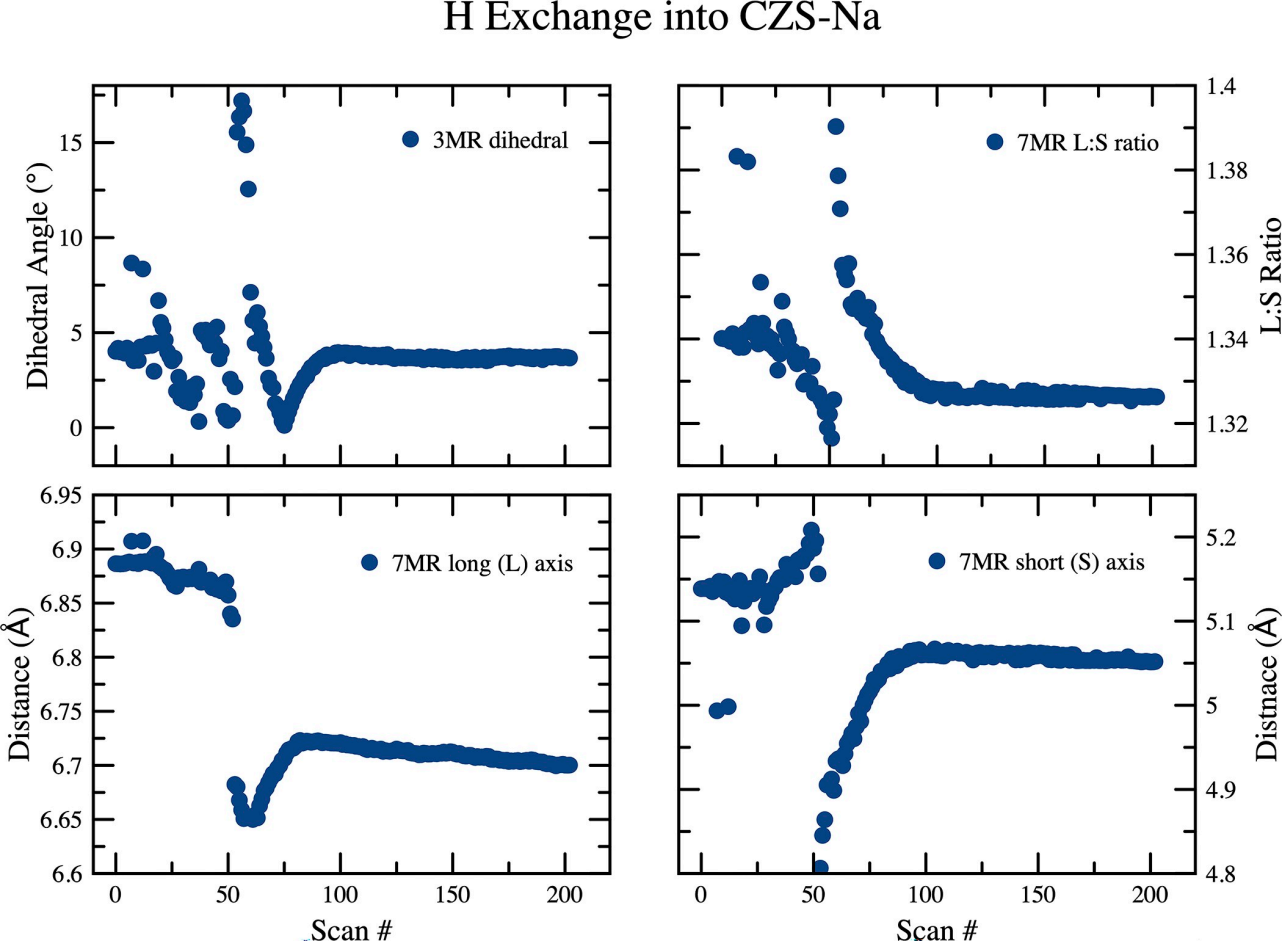
Plots of the 7MR and 3MR geometry for the H exchange into CZS-Na. Data points are larger than calculated error values.

The H^+^ exchange into CZS-Na occurred in two distinct steps (Fig 8). The first step can be seen in the unit-cell refinement plots to occur between scan 0 and ≈45 (first 22 min), followed by rapid unit-cell changes. This is also seen in the 7MR and 3MR geometry changes (Fig 11) as major discontinuities in their trends. At scan 56, the dihedral angle reached a maximum of nearly 20°, and finally settled to approximately the same value as the beginning of the experiment of ≈4.5°. A similar discontinuity can be seen in the 7MR L:S ratio (Fig 11) where the ring goes toward circularity and then moved toward ellipticity at scan 56 (time 28 min), and ultimately achieving an L:S nearly the same as the start of the experiment of ≈1.33.

**Fig 12:**
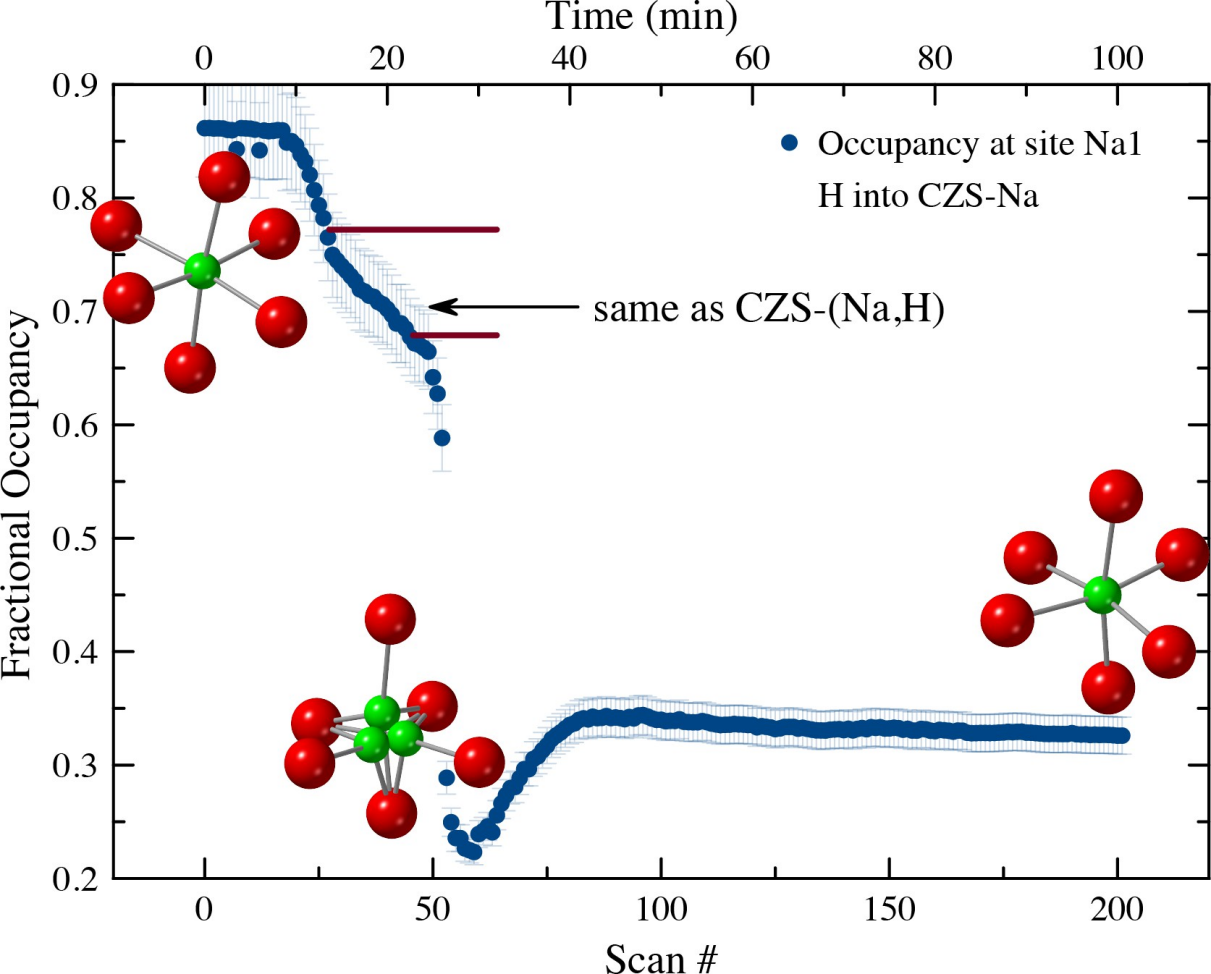
The occupancy values at site Na1 showed a stepwise decline in occupancy values until scan 56. After frame 56 the occupancy values increase. This increase is likely a result of H_2_O or H_3_O substitution into the Na1 site. TGA and DSC data (see supplementary information) confirm the higher hydration content. Na occupancy near the indicated arrow matches that for commercial CZS-(Na,H), before a rapid loss of Na^+^ during the transition area between the horizontal red marker lines. Insert ZrO_6_ models for the beginning (scan 1), point of highest Zr disorder correlating to highest 7MR and 3MR distortion (scan 56), and final structure (scan 200).

**Fig 13:**
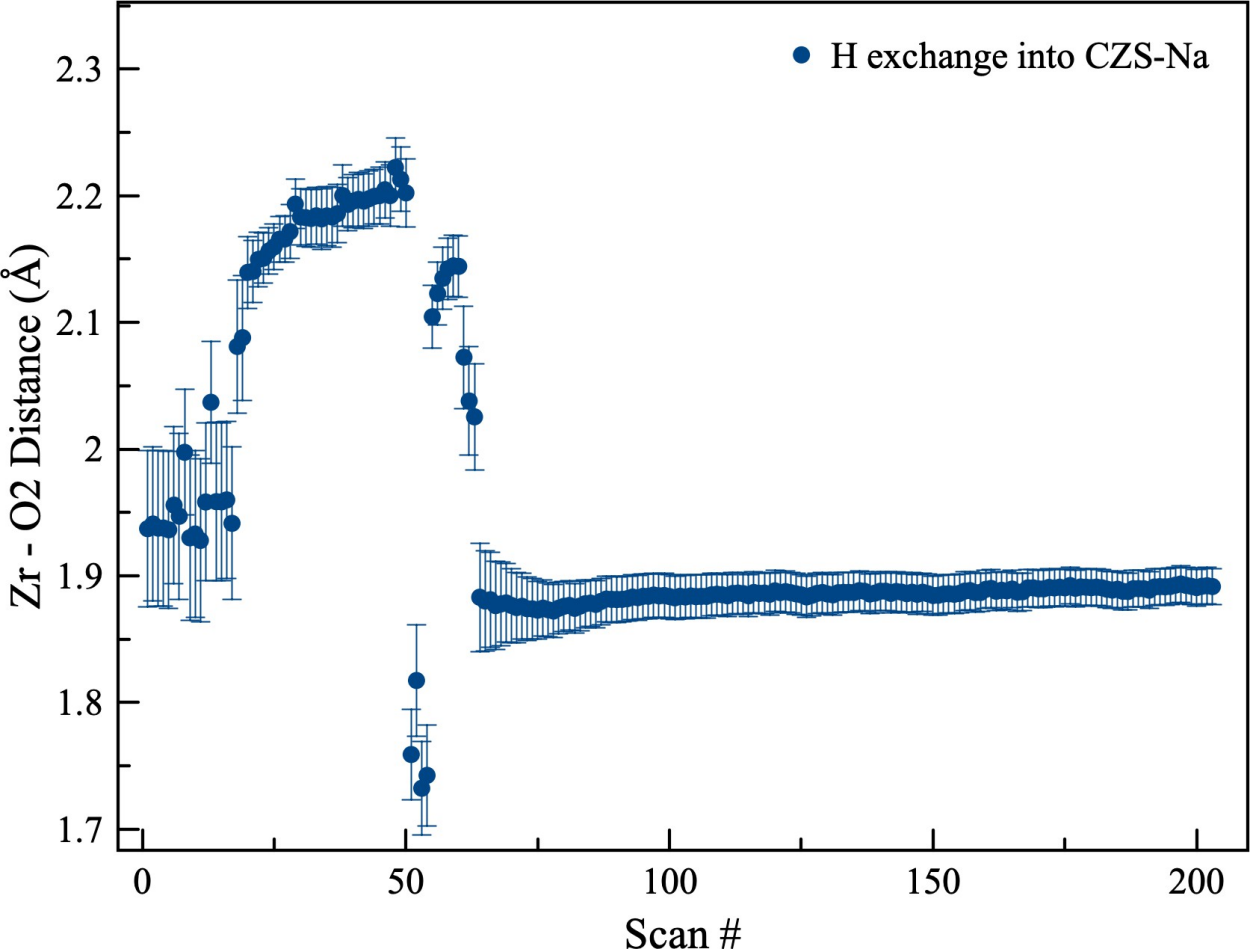
Plot of the Zr-O2 distances during H exchange into CZS-Na.

The beginning and final structures for the CZS-Na and CZS-H materials are not appreciable different (Fig 9), however there are major differences observed in the intermediate structures. At scan 56 (Figs 8 and 11) the ring ratio is at the highest ellipticity value. This eccentricity may be the cause of the stereo-selectivity for the high H^+^ of CZS materials. At scans less than 56, the H^+^ content is not as high, and the ring are more circular. At scans higher than 56, the high H^+^ content increases, but the rings become more circular. It was not possible to test the long-term structural stability of the crystal structure at scan 56. It is likely that this structure is transient, and over time the H^+^ on the O1 site would disperse through the channels. The greatest degree of structural distortion occurs at scan 56, where Zr-O2 bond is 1.65Å, Zr-O1 is 2.3Å, the 3MR dihedral is ≈20°, and the L:S ring ratio is ≈1.4. In addition, the occupancy at site Na1 is at the lowest value of 0.21 (Fig 12). The extent of this distortion cannot be fully explained from either Raman or XRD data. It is likely that the rapid increase in Bronsted acid sites (i.e. the OH groups on the O1 site) is forcing these structural distortions by shortening the Zr-O1 and lengthening the Zr-O2 (Fig 13), which in turn forces the 3MR and 7MR geometry changes. In addition to structural geometry changes, the Zr site split forcing long and short Zr-O bond lengths. Further neutron scattering and FTIR investigations would be needed to determine the extent of H_2_O or H_3_O^+^ substitution into the structure. This splitting only occurs between scan 45 and 85, and the Zr site recovers its starting position at the end of this experiment. It is remarkable to note that the crystal structure at scan 40, prior to splitting of the Zr site and structural distortion, has the same unit-cell volume (1074 Å^3^) and a Na1 occupancy of 0.7, which are all similar to the CZS-(Na,H) (the commercial form) crystal structure refined in this study.

### 3.3. K exchange into CZS-H

The exchange of K^+^ into the maximal H exchanged form (CZS-H) was also rapid. The Raman spectra showed signs of increased signal-to-noise, narrowing of FWHM for v5, v8, and the appearance of the lattice vibrations as K^+^ exchange proceeded. The ion exchange of K^+^ into CZS-H occurred in two distinct steps (see Fig 6). The first step was a rapid shift (6 as black line) of the v5 peak from 522.3 cm^-1^ to 525.7 cm^-1^ occurring over 10 minutes, where the latter peak position is close to the starting value of the CZS-Na peak. The second step in the ion exchange proceeded with a slow increase of the v5 peak from 525.7 cm^-1^ to 526.7 cm^-1^ over a 260 minute timeframe. In comparison with previous studies in zirconium silicate and titanium silicate materials, this two-step exchange process has been observed [18, 20] and attributed to be the result of cation migration in the channels. In the case of umbite, a microporous titanium silicate, K^+^ quickly exchanged into the channels, and then slowly migrates into the smaller intersecting tunnels [18]. This two-step process was confirmed by the time-resolved XRD study of K into CZS-H and CZS-Na (see Fig 8).

**Fig 14:**
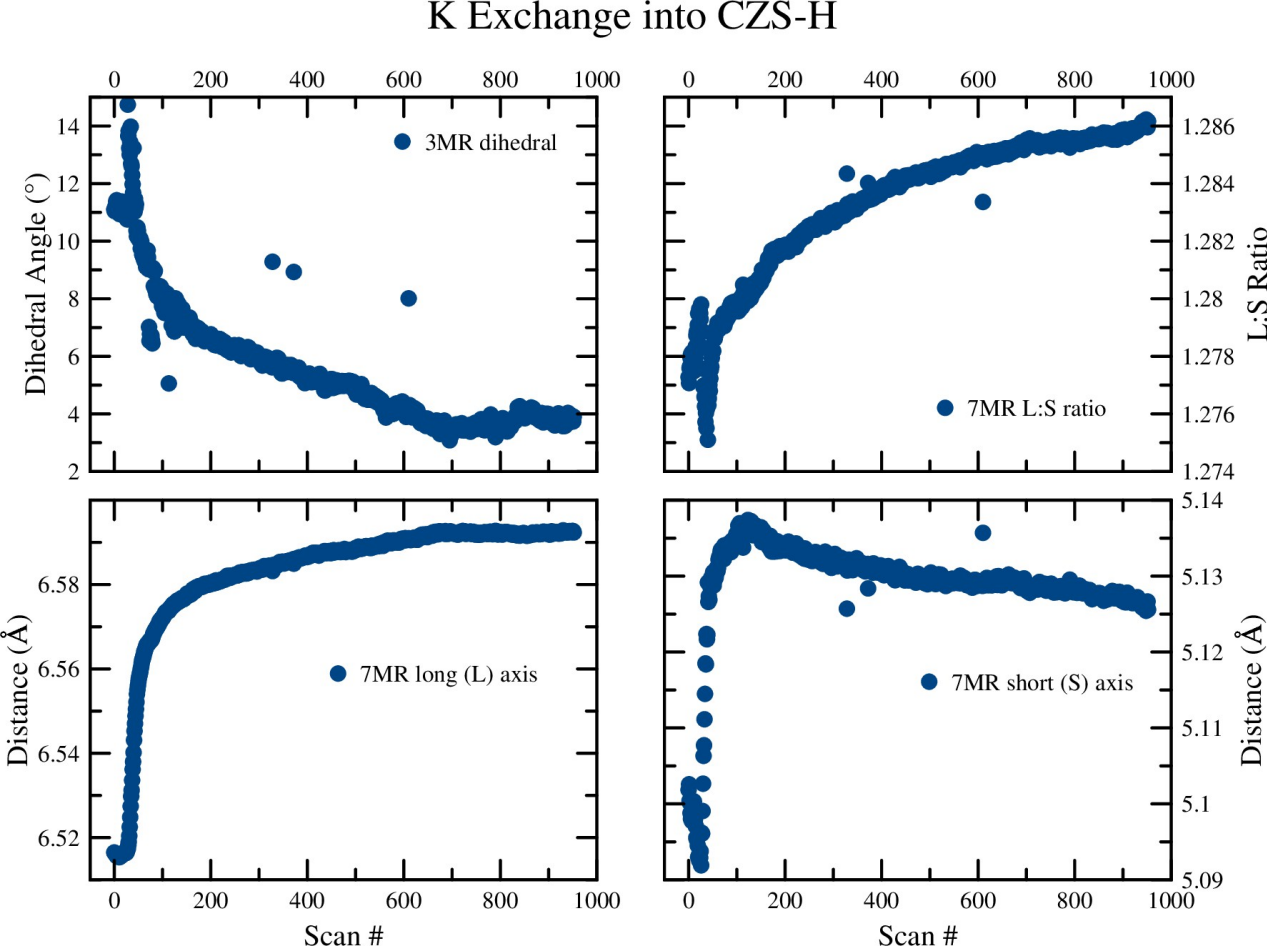
Plots of the 7MR and 3MR geometry for the K^+^ exchange into CZS-H. Data points are larger than calculated error values.

**Fig 15:**
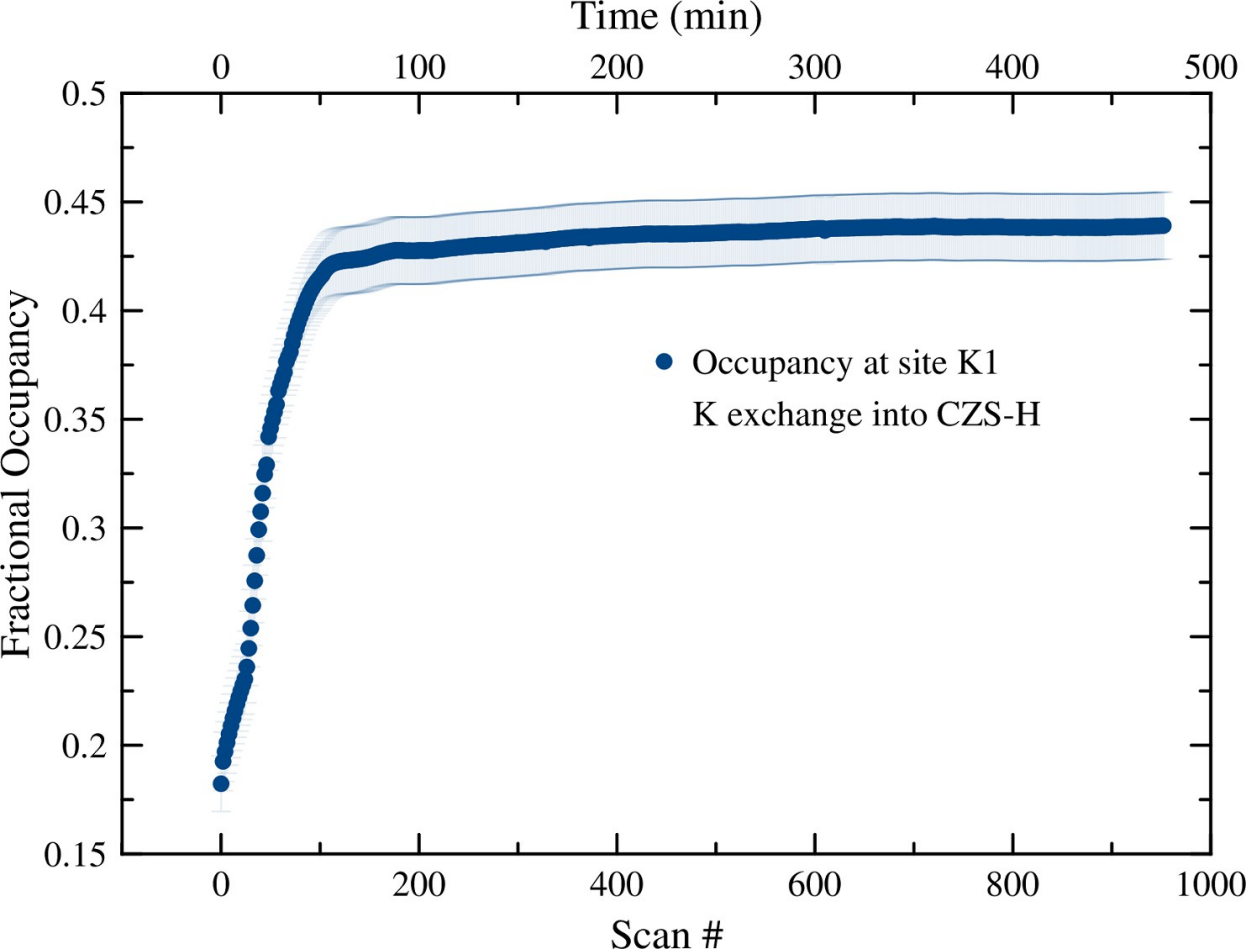
Plot of K1 occupancy during the K^+^ exchange into CZS-H. Data points are larger than calculated error values in GSAS-II. True errors are likely much larger (≈ ±0.03) because of OW2, Na1, and K1 overlapping sites that complicate occupancy calculations without independent chemical measurements.

After the H^+^ exchange experiment into CZS-Na was completed, the solution was changed to a 0.01M KCl. Crystal structures of the endmembers and 7MR/3MR geometry are shown in Fig 14. The occupancy of K^+^ increased rapidly from the onset of the ion exchange (to 0.4, see Fig 15). This K1 site is disordered with H_2_O (OW2) and Na1, and thus it was difficult to quantify the Na/H_2_O contribution to the total site occupancy. An occupancy constraint of Na1 + K1 = 1 was implemented to stabilize refinements, however, it was still difficult to quantify precise K1 occupancy without independent chemical measurements. The K1 occupancy trend closely matched that with the unit-cell axial decrease, dihedral angle change, and 7MR long axis length change, as well as the peak intensity ratio for 021:041 peaks (see supplementary information).

### 3.4. K exchange into CZS-(Na,H)

The peak fitting results of the time-resolved Raman spectroscopy for the v5 and v8 peaks are shown in Figs 6 and 7, respectively, and in Table 2. The starting position for the v5 peak of CZS-(Na,H) is 38(3)% between the CZS-Na and CZS-H starting values, as expected for CZS-(Na,H) being a partially protonated material. The rate of K ion exchange into CZS-(Na,H) was significantly slower as indicated by the hill slope value in Table 2. There appeared to be one ion exchange step as the K cation diffused into the CZS-(Na,H) structure.

Similar peak movement for the K into CZS-(Na,H) was observed to those of K into CZS-Na (See Figs 6 and 7), albeit rates of peak movement differ.

**Fig 16:**
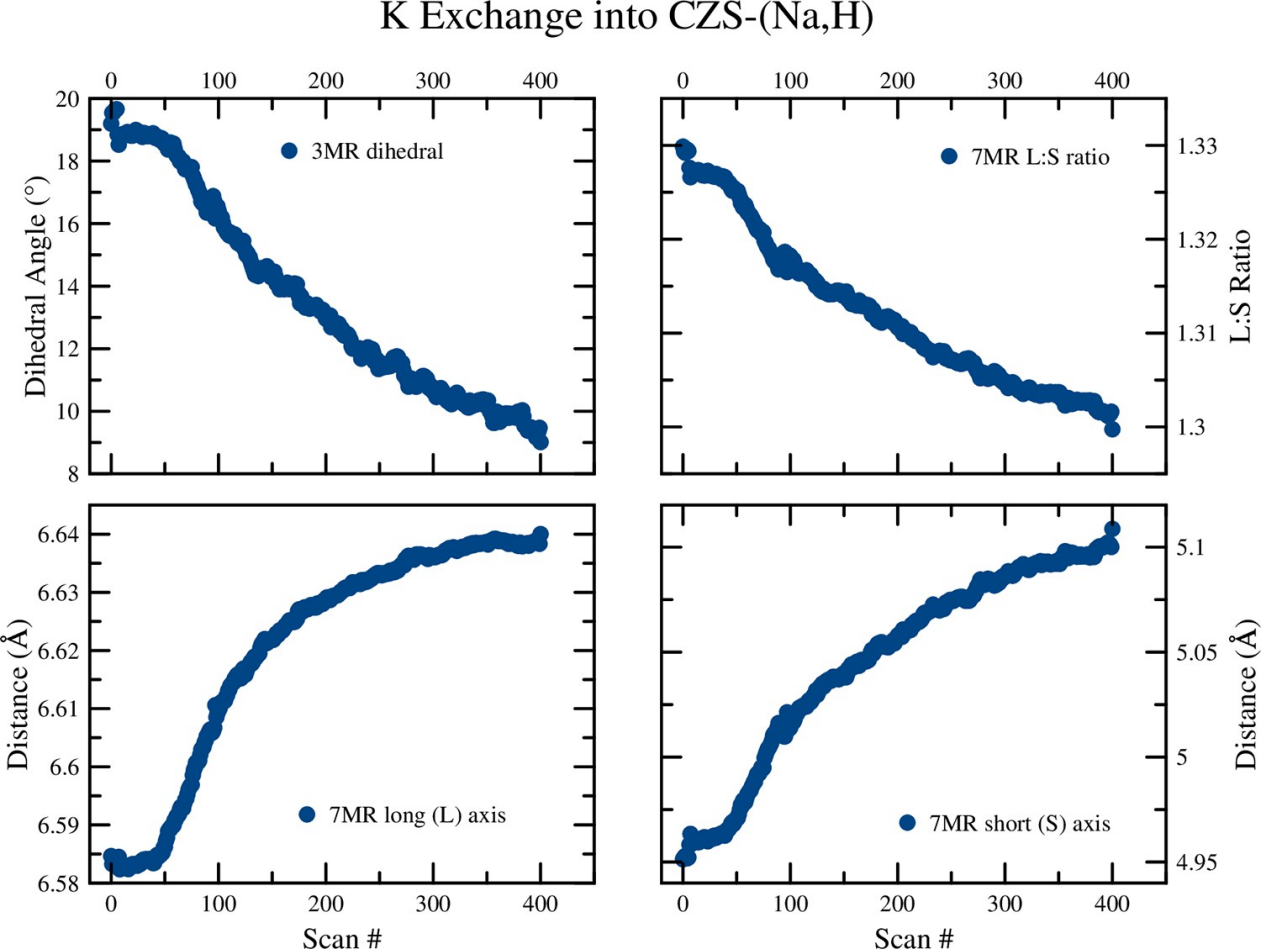
Plots of the 7MR and 3MR geometry for the K exchange into CZS-H. Data points are larger than calculated error values.

The changes observed in the unit-cell volume (Fig 8), 7MR ring geometry, and 3MR dihedral angle (Fig 16) all suggest a continuous diffusion process of K^+^ exchange into CZS(Na,H). Continuous refinement of the overlapping Na1, K1, and Ow2 sites prevented stable occupancy refinements during sequential Rietveld crystal structure refinements. Separately, static XRD refinements on a subset of data was performed to model K occupancy with greater precision. Splitting of the Zr site was not observed during the sequential refinements during K exchange. The unit-cell and 7MR became larger during ion exchange, and the ring ratio became more circular. In addition, the 3MR dihedral becomes more flat and reached a minimum values of 1.3° at the end of the experiment. This exchange experiment was the least dynamic in terms of rate of exchange and the absences of discontinuities in structure/ring geometry.

### 3.5. Back exchange experiments

**Fig 17:**
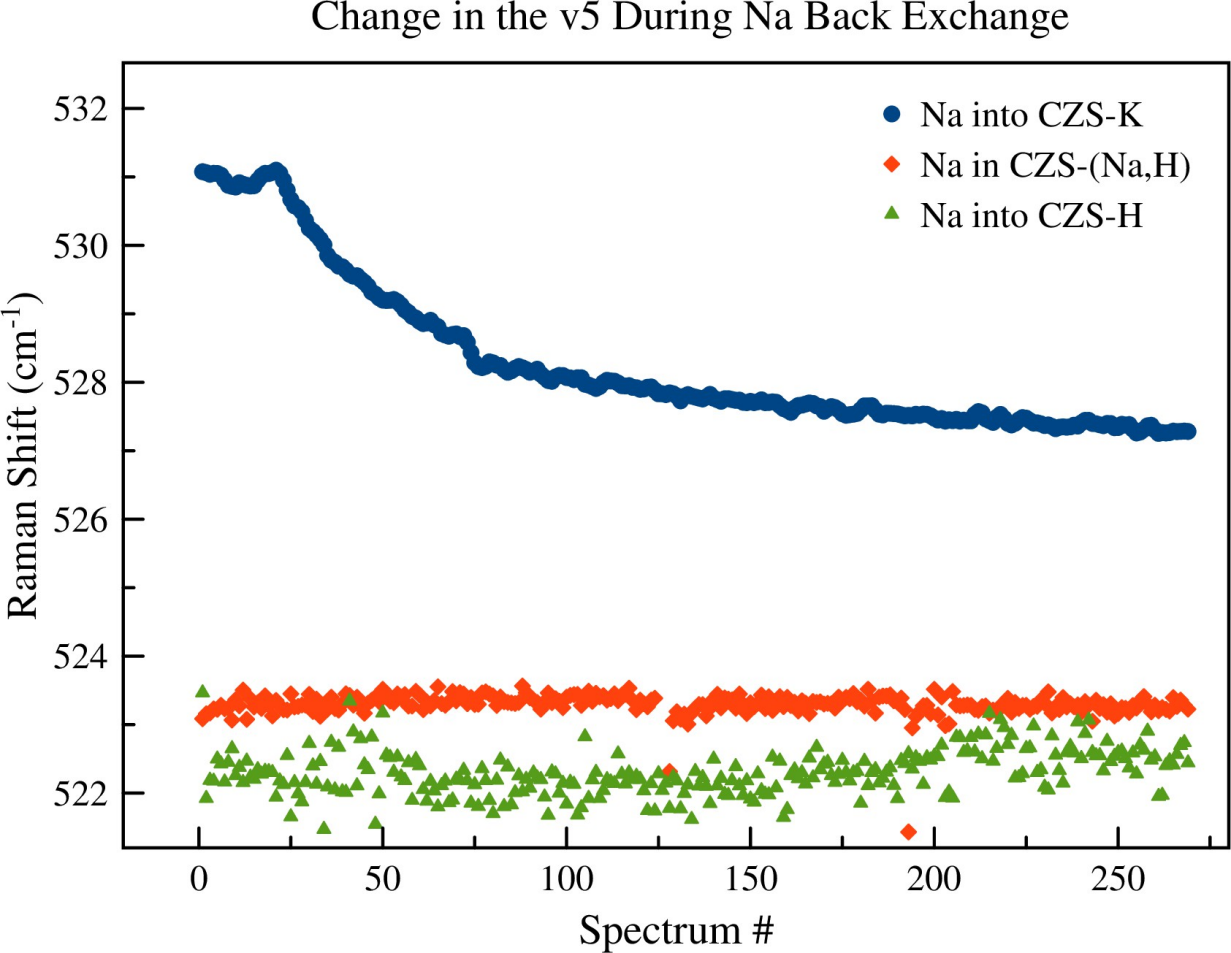
Results of the v5 peak fitting for time-resolved Raman spectroscopy studies for Na^+^ back exchange into CZS-K, CZS-(Na,H), and CZS-H. As observed from these plots, Na back exchange was only measurable for the CZS-K material. The v8 peak not shown for clarity, but showed similar trends as the v5 peak.

To better quantify the role of H^+^ on the ion exchange mechanisms, time-resolved Raman spectroscopy ion exchange experiments were performed using 0.1 M NaOH into CZS-H, CZSK, and CZS-(Na,H). This high concentration of NaOH was an attempt to achieve high Na ion exchange strength and Donnan potential [29] to force possible ion exchange in a reasonable timeframe. In these experiments (Fig 17), only a slight peak movement (1 cm^-1^) of the v5 and v8 peaks for the Na^+^ into CZS-(Na,H) and CZS-H were observed. Significant peak movement of the v5 and v8 modes for the Na^+^ exchange into CZS-K (i.e., when there was no OH groups on the framework) were measured. Time-resolved XRD experiments using the same conditions as the Raman studies were performed. The limited availability of synchrotron radiation for this study did not allow for long term experiments, however, time-resolved XRD data also showed no measurable changes in unit-cell volume for the Na^+^ exchange into CZS-(Na,H) or CZS-H. From this, it was concluded that Na back-exchange in the CZS-(Na,H) and CZS-H materials is not favorable.

## 4. Discussion

### 4.1. Controlling parameters for ion exchange

The controlling parameters for ion exchange in natural zeolites and zeolite-like materials are determined on a case-by-case basis. There are no known established chemical or physical laws that govern the ion exchange properties of these microporous materials. That said, there are physiochemical properties that can be investigated to better understand what the ion exchange controls are on ion conductivity through the micropores, and those controls primarily being pore/ring, channel geometry and dimension, framework geometry/composition/flexibility, host cation type and crystallographic location, guest cation type and crystallographic location, and extent of hydration and acidity (i.e. number of H_2_O and OH in the channels, respectively).

Without using a crystal structure model to describe the ion exchange mechanisms, the time-resolved Raman spectra may be used to quickly measure rates and extent of cation exchange in the CZS materials. If the relative percent Raman shift for the v5 peak is an indicator for extent of ion exchange, then taking the difference of the total range of the v5 value would yield the total ion exchange extent for K^+^. The range of v5 for the K^+^ exchange into CZS-Na is 3.4 cm^-1^ taking 19 min, K^+^ exchange into CZS-H is 3.4 cm^-1^ taking 10 min (not accounting for the 1 cm^-1^ shift in the v5 peak during the second exchange step), and approximate 200 min for K^+^ exchange into CZS-(Na,H). Since the difference in values of the v5 peak are the same for both the K^+^ into CZS-Na and CZS-H, then total exchange could be identical. However, this has not been verified by chemical analysis. Similar changes in the magnitude for the range of v8 are also observed.

If the absolute value of Raman shift for the v5 peak is the indicator for extent of K^+^ exchange in CZS-(Na,H), CZS-Na, and CZS-H, then CZS-H is the poorest performer for K^+^ exchange (thus having the lowest cation exchange capacity), followed by CZS-(Na,H), and CZS-Na having highest cation exchange capacity. CZS-(Na,H) may have slightly lower cation exchange capacity as indicated by the maximal value of the v5 peak as compared to the maximal value of the v5 peak for CZS-Na (see Fig 6). It should be noted that this model also places CZS-H as being the fastest exchanger during initial uptake, and then significantly slows after the rapid first exchange step (see Fig 6).

### 4.2. Extent of ion exchange as modeled via X-ray diffraction

The 3MR torsion angle (a relative measure of the degree of distortion) increases as a function of K^+^ exchange depending on the host cation type. When the compound starts in the as synthesized Na-from (CZS-Na) and exchanging into the K-form (CZS-(Na,K)), the torsion angle changes less than 1°. As H^+^ content increases, the difference in the torsion angle between the starting material and the final ion exchange K-form increases. The greater the torsion angle, the greater the twisting of the 3MR, and this was observed in the large Raman shifts for v5 peak. In the CZS materials, there is no known correlation between 3MR torsion angle and structural stability. The torsion angle was used as a measure of cation exchange extent and as a measure of the exchange mechanisms.

The coordination geometry of K−O^2–^, H_2_O, as modeled from X-ray diffraction data, also changed depending on the host material starting composition. The contribution of K^+^ toward charge balance was calculated using the BVS model (bond valence sum, see supplementary information), where the BVS should be unity in the case of K^+^. The structure with the most ideal BVS was the K exchanged CZS-(Na,H), with a BVS of 0.93 v.u.

Although good models of the ion exchange mechanisms have been determined, the precise role of the H bonding network cannot be completely evaluated as the H^+^ positions on the framework or interstitial H_2_O molecules could not be found experimentally measured this powder X-ray diffraction study. Future work using neutron scattering with deuterated forms of the starting material would need to be performed.

## 5. Conclusions

Based on joint XRD, Raman, and TGA/DSC (see supplementary information) the ion exchange mechanisms for the CZS materials was related to the hydroxyl and hydration state in CZS as potentially involving similar mechanisms as seen in protein ion channels and microporous titanium silicates [19, 30, 31]. In those studies, ion selectivity is related to both the relative dimensions of the cation-to-ring sizes and also the in-going cation-hydrogen interaction. In the case of microporous titanium silicates, the ion selectivity involved two steps: the ingoing cation had to hydrate with H_2_O in the channels, and this hydration forced the H_2_O closer to the hydroxyls on the framework. This newly formed H_2_O…OH interaction forced a conformational change in the framework to allow the cations to pass through the channels.

**Fig 18:**
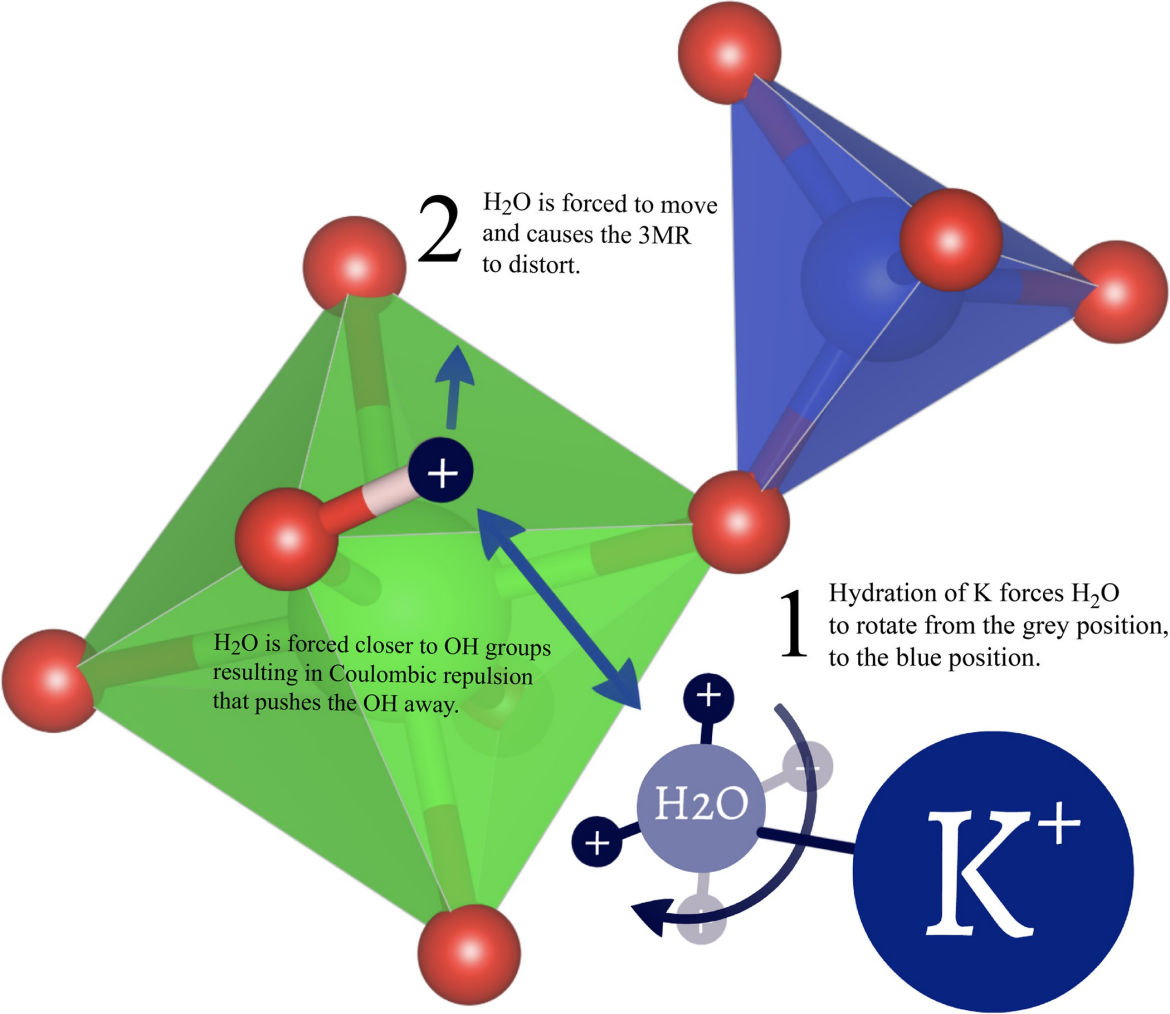
An illustration of part of a 3MR showing how the double lever mechanisms works in CZS-(Na,H). The first lever (labeled as ‘1’) has the K^+^ entering the crystal structure and interacting with H^+^ on the H_2_O (grey H sites) as K^+^ hydrates in the 7MR channels. The induced charge repulsion between the in-going K^+^ and H^+^ forces the H_2_O to rotate away, and the new H_2_O configuration (dark blue H^+^) now interacts closely with the framework. The second lever (labeled as ‘2’) forces the 3MR dihedral angle to increase (see Table 3) as the H^+^ on the newly rotated H_2_O forces the OH^−^on the ZrO_6_ to repel. Based on our XRD and Raman measurements, it is posited that this is the controlling factor during the ion selectively process. K^+^ has a lower hydration energy than sodium (-321 kJ/mol vs -405 kJ/mol) [32].

In the absence of neutron scattering data, and with molecular and crystallographic models of CZS depicting probable locations of the OH as proposed by Kan and Clearfield [11] (see Fig 2) and from BVS calculations in this study (see supplementary information), a few models can be proposed to satisfy currently collected data for the ion exchange mechanism in CZS materials. A comparative analysis for all time-resolved ion exchange experiments performed thus far are as follows:

- Na^+^ is all but excluded in the Na^+^ exchange into CZS-H or CZS-(Na,H) ion exchange experiment, while K^+^ into CZS-H ion exchange proceeded rapidly
- Na^+^ can slowly exchange back CZS-(Na,K) material
- K^+^ was the only cation of the three tested (H, Na, K) to exchange into the CZS-Na, CZS-(Na,H), or CZS-H materials that result in a positive Raman shift (blue shift) for the v5 peak, indicating stronger interstitial cation-framework interaction with the 3MR.
- H^+^ and K^+^ can exchange into CZS-Na and CZS-(Na,H).
- From the above list, it is clear that the role of H^+^ in CZS-(Na,H) has a large influence over cation exchange selectivity and capacity when temperature and pressure are left invariant. Previous studies [17, 19] have suggested that the H_2_O…OH network is very different in the H^+^ form of microporous material versus when larger cations (Li, Na, K, Cs etc.) occupy the channels. In those previous studies, the H-bond network was determined by neutron scattering data in a titanium silicate [17], and was shown to only change hydrogen-bond geometry when a cation of a certain type entered the framework. The forcing behind the hydrogen bond network change was caused by hydration of in-going cation (in that case Cs). That selective hydration process allowed the ion exchange of the cation to take place, and was termed the ‘double-lever’ mechanisms, as a reference to its striking similarity to the ‘paddle’ mechanisms found in protein ion channels [31]. It is theorize from these observations that a similar double-lever mechanism is working in the CZS materials (Fig 18), and neutron diffraction studies are being planned for CZS suite of materials.

## Supporting information

S1 Table ZrO_6_ bond valance sum calculations from VESTA in v.u. (DOCX)

S2 Table Description of Raman studies and instrument settings. (DOCX)

S1 Fig Time-resolved Raman data contour plot for the K into CZS-Na. (TIF)

S2 Fig Time-resolved Raman data contour plot for the H into CZS-Na. (TIF)

S3 Fig Time-resolved Raman data contour plot for the K into CZS-NaH. (TIF)

S4 Fig Time-resolved Raman data contour plot for the K into CZS-H. (TIF)

S5 Fig Example of peak fit for CZS-Na. Example peak fitting for CZS-Na. Raw data in black shown without background profile fitting. Fitted peaks are for background subtracted Raman spectra. Bold dashed red line is the profile sum-curve. Background shown as blue dashed line.
(TIF)

S6 Fig Thermogravimetric of CZS-Na. TGA data shown in green, DSC data in blue. Data were collected using a Netzsch STA 449 F1 Jupiter with using a constant temperature ramp of 5°C/min. Data shows two endothermic weight losses attributed to water loss.
(TIF)

S7 Fig Thermogravimetric analysis and differential scanning calorimetry of CZS-H. TGA data shown in green, DSC data in blue. Data were collected using a Netzsch STA 449 F1 Jupiter with using a constant temperature ramp of 5°C/min. Data shows two endothermic weight losses attributed to water loss.
(TIF)

S8 Fig Thermogravimetric analysis and differential scanning calorimetry of CZS-NaH. TGA data shown in green, DSC data in blue. Data were collected using a Netzsch STA 449 F1 Jupiter with using a constant temperature ramp of 5°C/min. Data shows only one endothermic weight losses attributed to water loss.
(TIF)

S9 Fig Simulated X-ray diffractograms for CZS with varying Na:H ratios. Calculated X-ray diffraction plots of various CZS Na/H ratios. Bottom pattern represents 100:0 (Na:H ratio) and top pattern represents 0:100 (Na:H ratio). The ratio of the two most intense peaks for CZS-Na (0 2 1) and (0 4 1) are shown in the insert. This plot was used to aid modeling of Na:H content during Rietveld refinements.
(TIF)

S10 Fig X-ray diffractograms for K exchanged forms of CZS. Plot of three diffraction patterns for the maximal H-exchanged form (green), maximal K-exchanged into the maximal H-exchanged form (orange), and maximal K-exchange into as-synthesized CZS-Na. This plot serves as an indicator to approximate the total amount of K-exchange into CZS materials, and this plot suggests that approximately 60% exchange into CZS materials (materials with high H-content) is a theoretical maximum, which was observed in our Rietveld refinements. Insert: orange line is the (021) (041) line from Fig 9 and blue line is the refined K content from synchrotron XRD data, that matches ‘best-guess’ as discussed above.
(TIF)

S11 Fig Change in peak intensity ratio of K into CZS-H. Evolution of the peak intensity ratio for the 041 and 021 during K exchange into CZS-H of data collected at the APS synchrotron.
(TIF)

S12 Fig Change in peak intensity ratio of H into CZS-Na. Evolution of the peak intensity ratio for the 041 and 021 during H exchange into CZS-Na of data collected at the APS synchrotron.
(TIF)

S1 Appendix All structures as CIFs. (CIF)
