# Supplementary material for: In situ potassium and hydrogen ion exchange into a cubic zirconium silicate microporous material
Source: PLoS One. 2024 Mar 21;19(3):e0298661. doi: 10.1371/journal.pone.0298661 (PMC10956793; doi:10.1371/journal.pone.0298661)
Supplement: S1 Table — (DOCX) [file pone.0298661.s001.docx]

**S1 Table. ZrO_6_ bond valance sum calculations from VESTA in v.u.**

| Bond valance parameter used | 1.928 |
| --- | --- |
| O1 | -0.634158 |
| O1 | -0.634157 |
| O1 | -0.634158 |
| O2 | -0.649002 |
| O2 | -0.649002 |
| O2 | -0.649002 |
| Bond valence sum | 3.849 |
| oxidation state of cation | +4 |
| Expected bond length | 2.078 ˚A |
