# Supplementary material for: In situ potassium and hydrogen ion exchange into a cubic zirconium silicate microporous material
Source: PLoS One. 2024 Mar 21;19(3):e0298661. doi: 10.1371/journal.pone.0298661 (PMC10956793; doi:10.1371/journal.pone.0298661)
Supplement: S2 Table — (DOCX) [file pone.0298661.s002.docx]

**S2 Table. ZrO_6_ bond valance sum calculations from VESTA in v.u.**

| Temperature | 24 ^◦^*C* |
| --- | --- |
| Ion Exchanger Electrolyte | 0.01M KCl, NaCl, or HCl in deionized H_2_O |
| Electrolyte Flow Rate | 0.05 mL/minute |
| Data Acquisition Times | Data collected continuously:  400 spectra, 30 sec. (10 sec. acquisitions 3x) |
| Laser | 780 nm, 14mW |
| Objective and slits | 10x and 50 m |
| Laser spot size | 2 m |
| Spectral resolution | 1 cm^–1^ |
