# Supplementary figures and images for: In situ potassium and hydrogen ion exchange into a cubic zirconium silicate microporous material
Source: PLoS One. 2024 Mar 21;19(3):e0298661. doi: 10.1371/journal.pone.0298661 (PMC10956793; doi:10.1371/journal.pone.0298661)

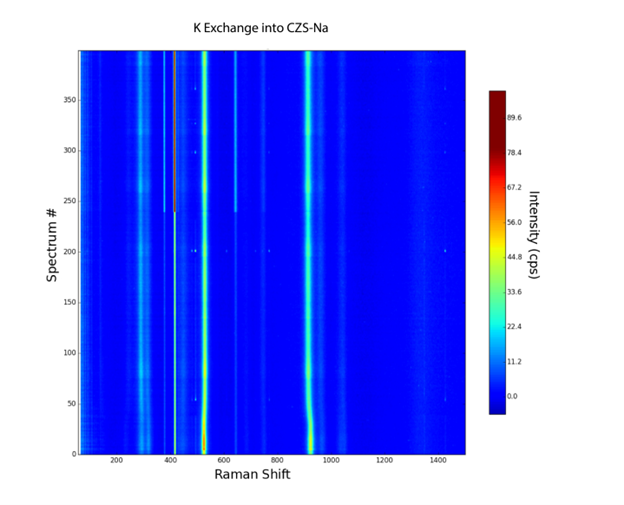

Supplement: S1 Fig — (TIF) [file pone.0298661.s003.tif]

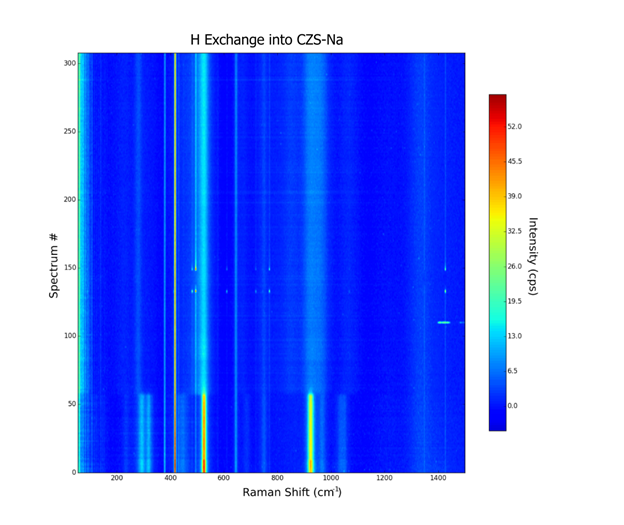

Supplement: S2 Fig — (TIF) [file pone.0298661.s004.tif]

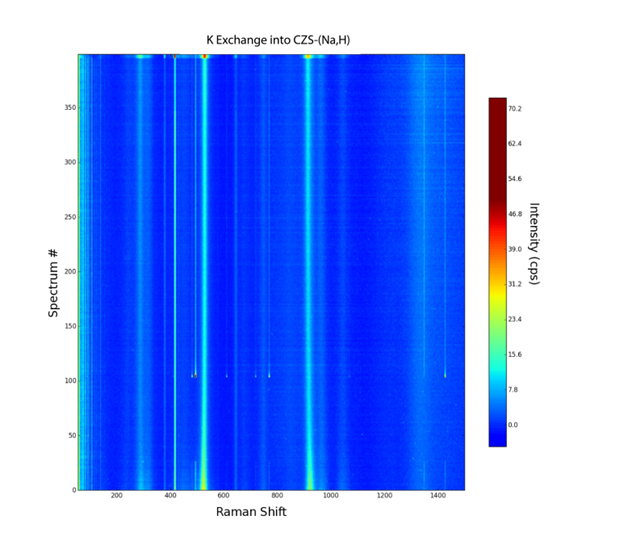

Supplement: S3 Fig — (TIF) [file pone.0298661.s005.tif]

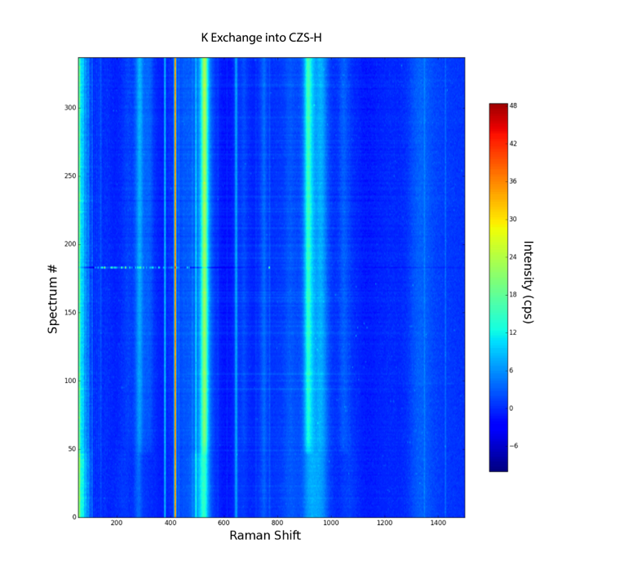

Supplement: S4 Fig — (TIF) [file pone.0298661.s006.tif]

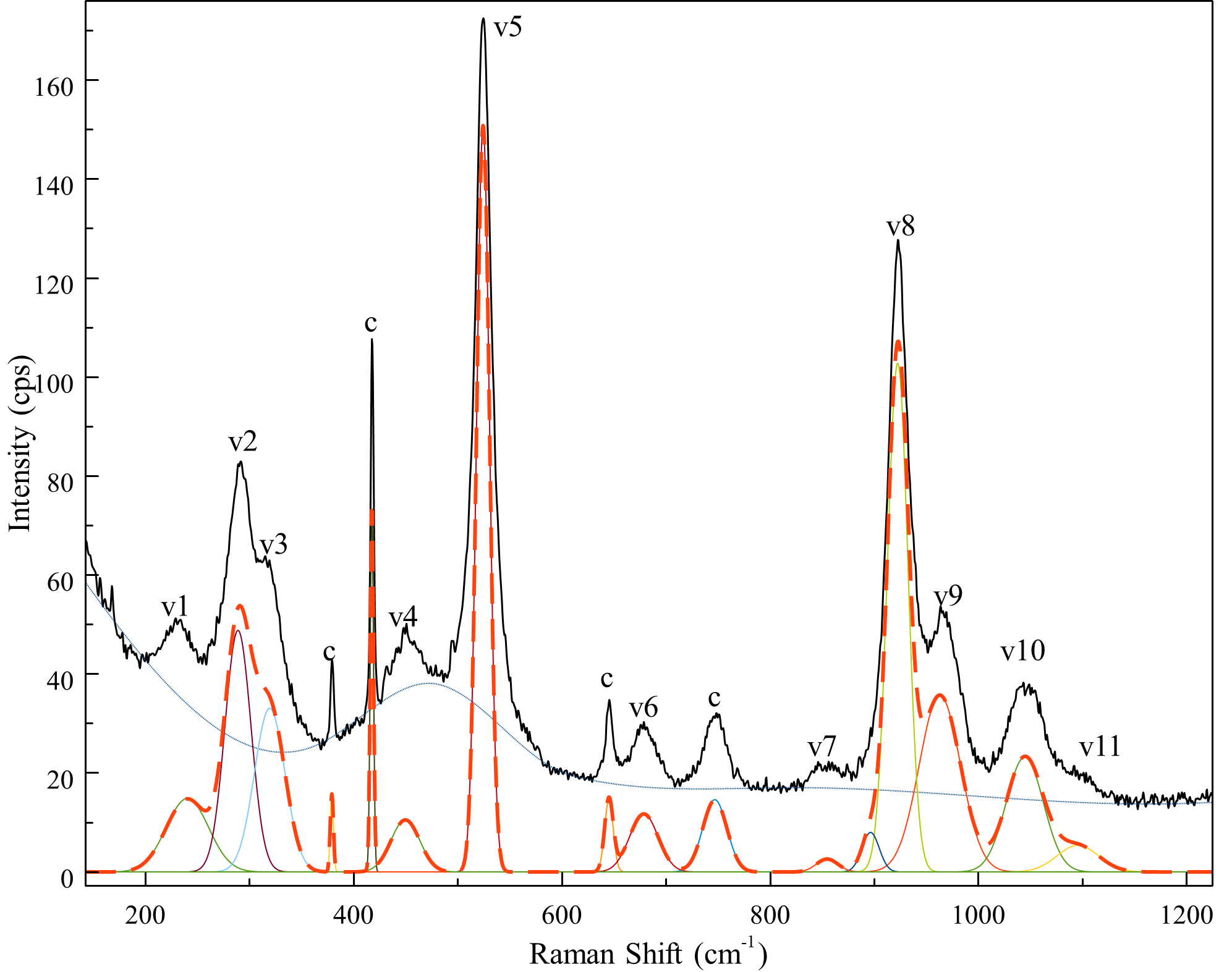

Supplement: S5 Fig — Example peak fitting for CZS-Na. Raw data in black shown without background profile fitting. Fitted peaks are for background subtracted Raman spectra. Bold dashed red line is the profile sum-curve. Background shown as blue dashed line. (TIF) [file pone.0298661.s007.tif]

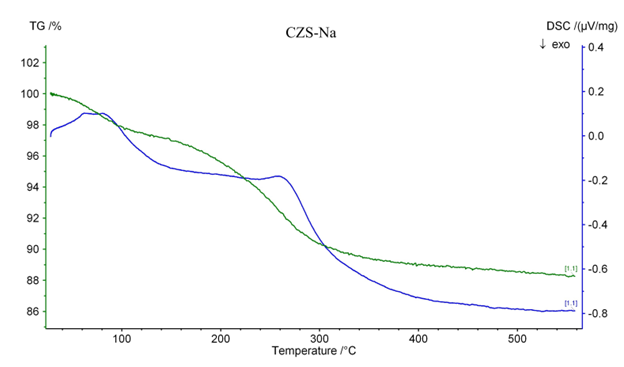

Supplement: S6 Fig — TGA data shown in green, DSC data in blue. Data were collected using a Netzsch STA 449 F1 Jupiter with using a constant temperature ramp of 5°C/min. Data shows two endothermic weight losses attributed to water loss. (TIF) [file pone.0298661.s008.tif]

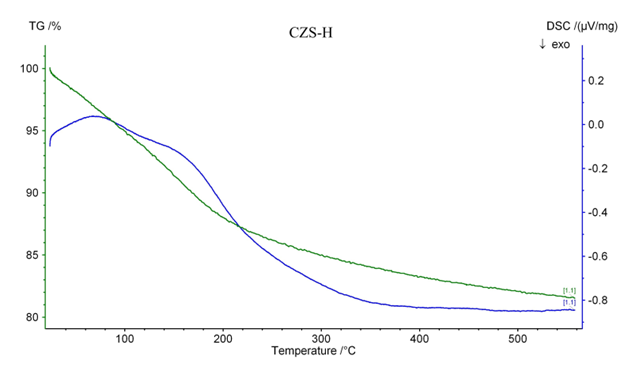

Supplement: S7 Fig — TGA data shown in green, DSC data in blue. Data were collected using a Netzsch STA 449 F1 Jupiter with using a constant temperature ramp of 5°C/min. Data shows two endothermic weight losses attributed to water loss. (TIF) [file pone.0298661.s009.tif]

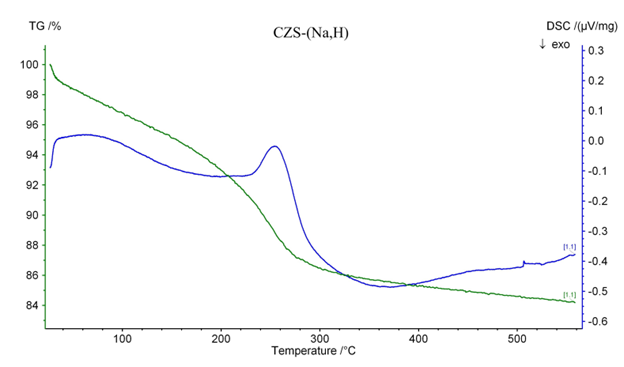

Supplement: S8 Fig — TGA data shown in green, DSC data in blue. Data were collected using a Netzsch STA 449 F1 Jupiter with using a constant temperature ramp of 5°C/min. Data shows only one endothermic weight losses attributed to water loss. (TIF) [file pone.0298661.s010.tif]

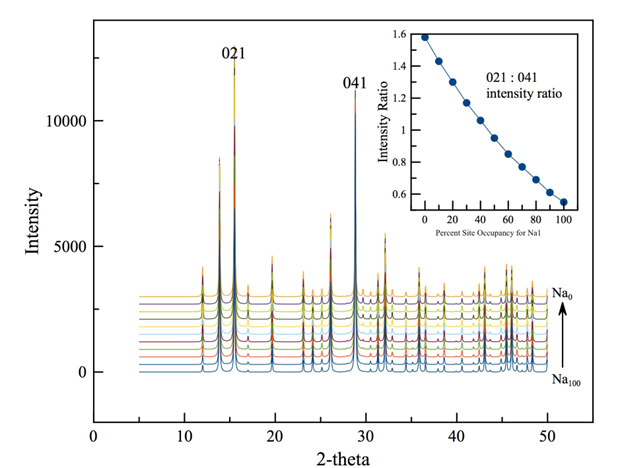

Supplement: S9 Fig — Calculated X-ray diffraction plots of various CZS Na/H ratios. Bottom pattern represents 100:0 (Na:H ratio) and top pattern represents 0:100 (Na:H ratio). The ratio of the two most intense peaks for CZS-Na (0 2 1) and (0 4 1) are shown in the insert. This plot was used to aid modeling of Na:H content during Rietveld refinements. (TIF) [file pone.0298661.s011.tif]

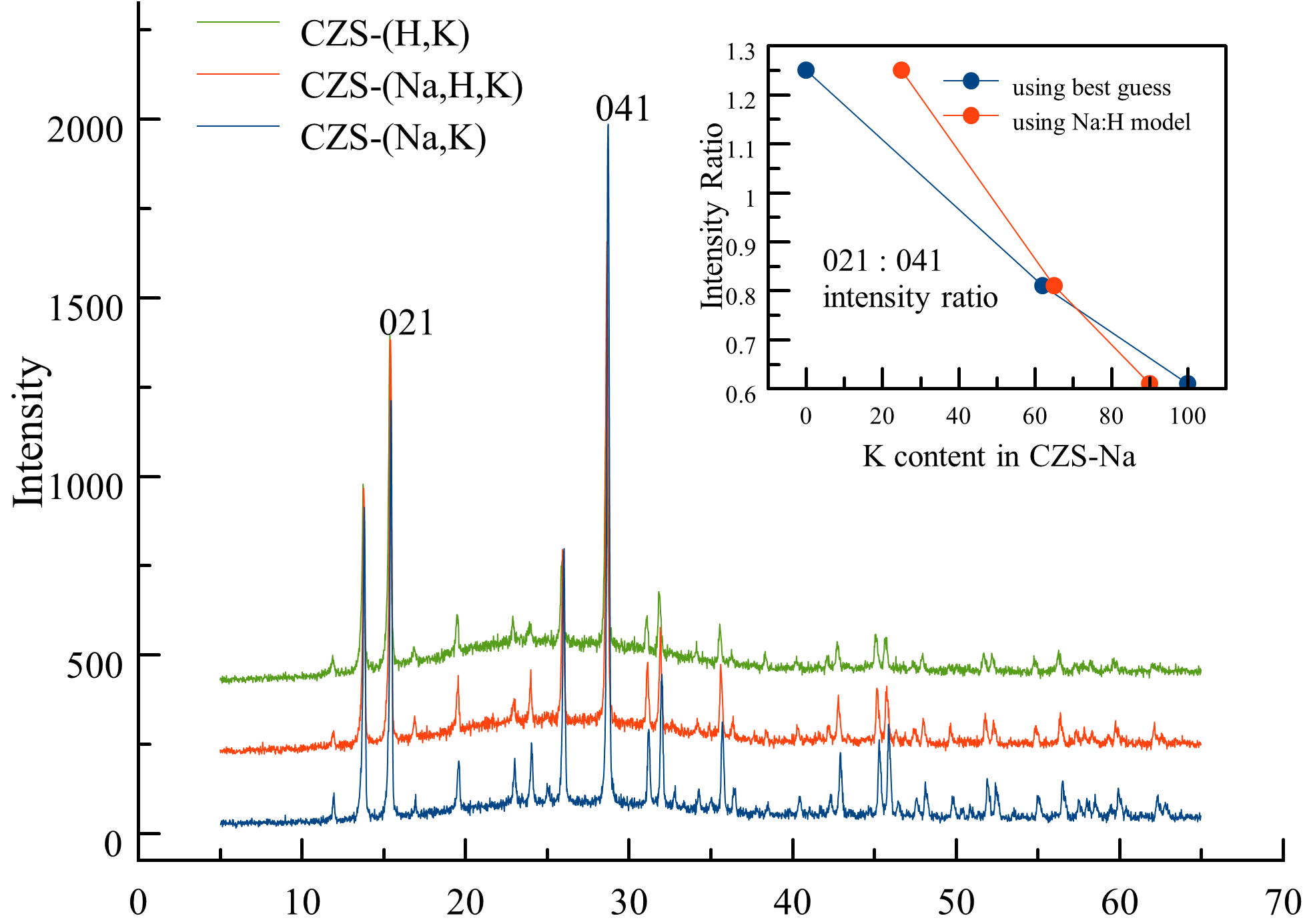

Supplement: S10 Fig — Plot of three diffraction patterns for the maximal H-exchanged form (green), maximal K-exchanged into the maximal H-exchanged form (orange), and maximal K-exchange into as-synthesized CZS-Na. This plot serves as an indicator to approximate the total amount of K-exchange into CZS materials, and this plot suggests that approximately 60% exchange into CZS materials (materials with high H-content) is a theoretical maximum, which was observed in our Rietveld refinements. Insert: orange line is the (021) (041) line from Fig 9 and blue line is the refined K content from synchrotron XRD data, that matches ‘best-guess’ as discussed above. (TIF) [file pone.0298661.s012.tif]

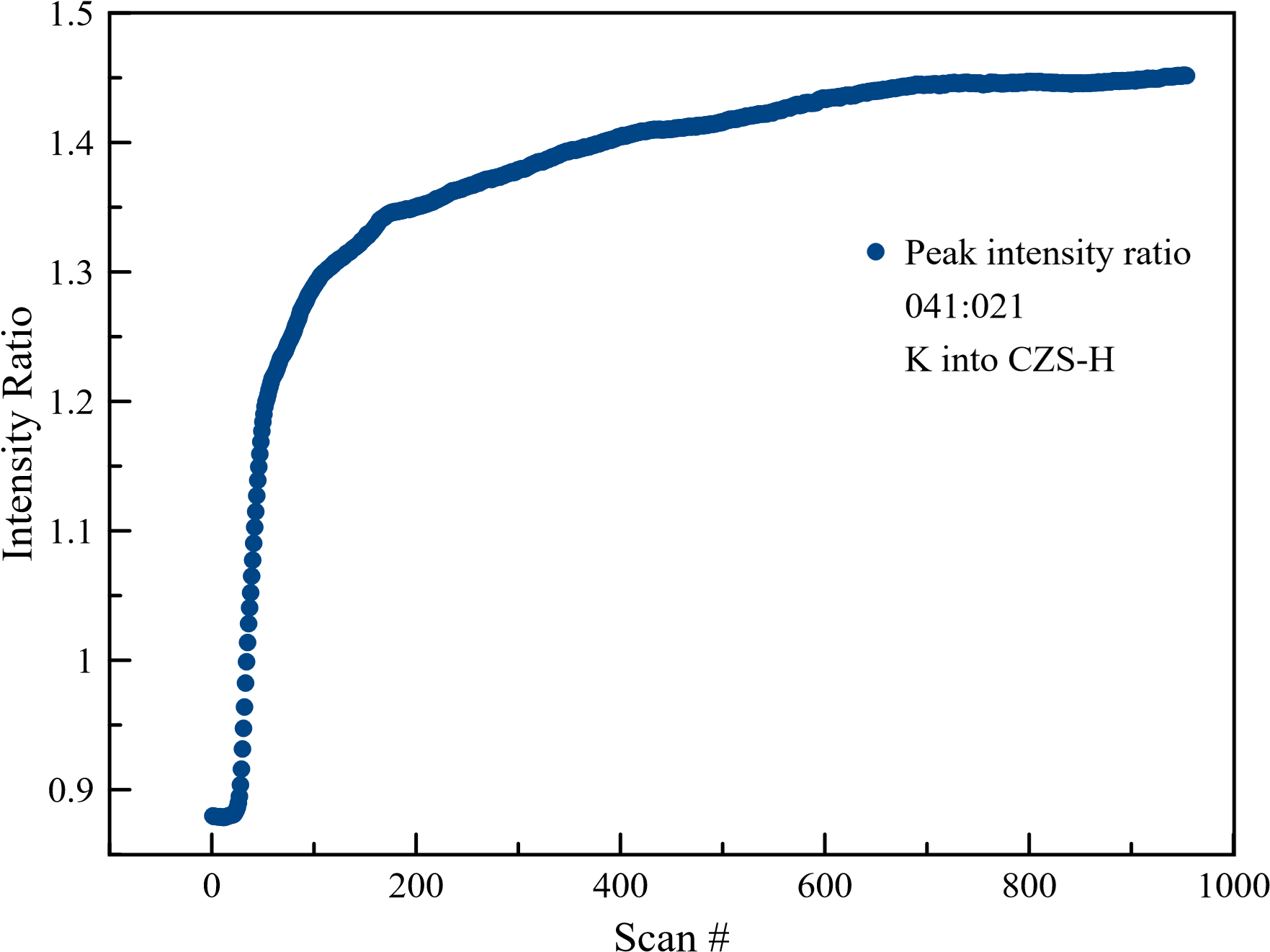

Supplement: S11 Fig — Evolution of the peak intensity ratio for the 041 and 021 during K exchange into CZS-H of data collected at the APS synchrotron. (TIF) [file pone.0298661.s013.tif]

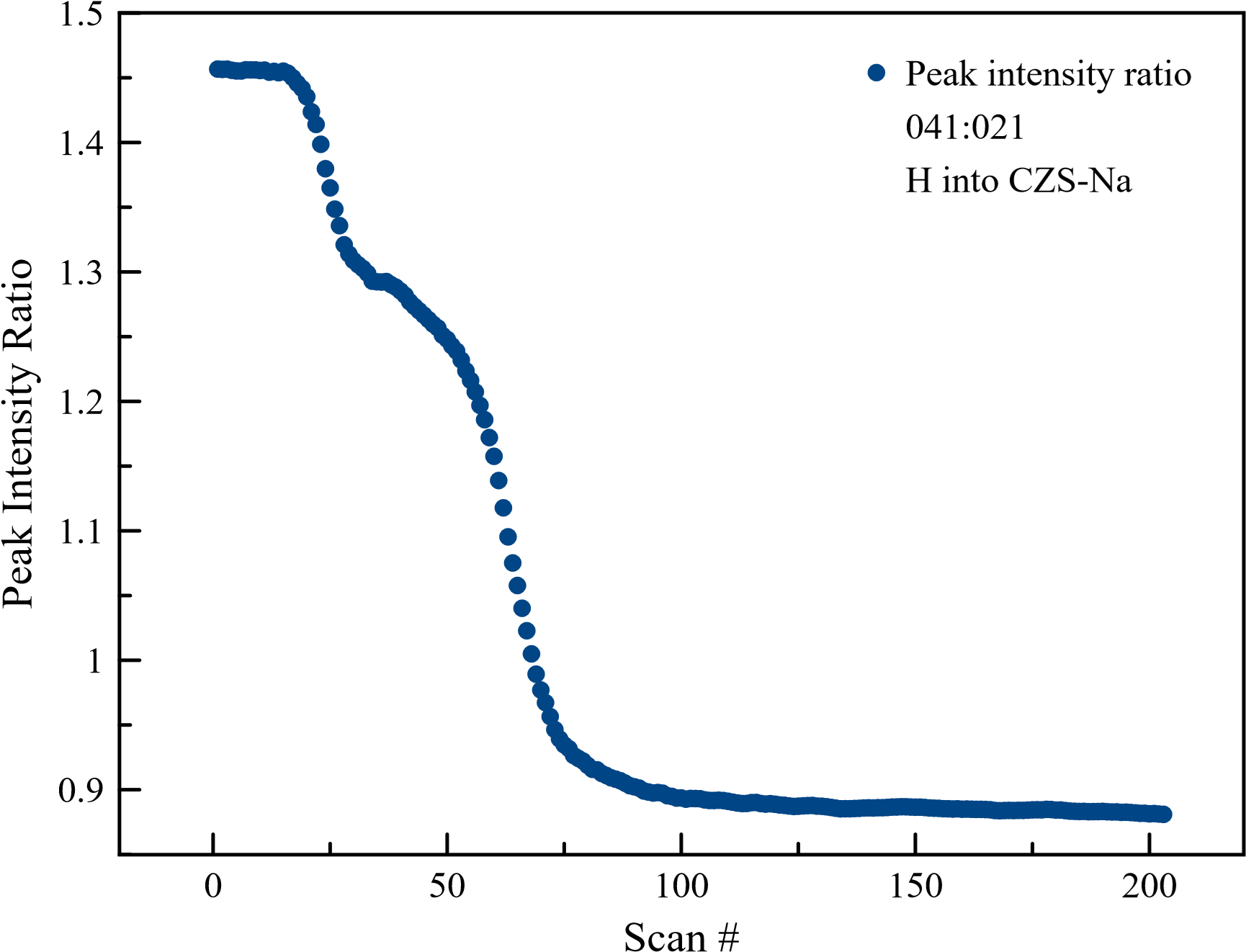

Supplement: S12 Fig — Evolution of the peak intensity ratio for the 041 and 021 during H exchange into CZS-Na of data collected at the APS synchrotron. (TIF) [file pone.0298661.s014.tif]
